# Chronic SSRI stimulation of astrocytic 5-HT_2B_ receptors change multiple gene expressions/editings and metabolism of glutamate, glucose and glycogen: a potential paradigm shift

**DOI:** 10.3389/fnbeh.2015.00025

**Published:** 2015-02-20

**Authors:** Leif Hertz, Douglas L. Rothman, Baoman Li, Liang Peng

**Affiliations:** ^1^Laboratory of Brain Metabolic Diseases, Institute of Metabolic Disease Research and Drug Development, China Medical UniversityShenyang, China; ^2^Magnetic Resonance Research Center, Diagnostic Radiology and Biomedical Engineering, Yale UniversityNew Haven, CT, USA

**Keywords:** calcium homeostasis, fluoxetine signaling, 5-HT_2_ receptors, glucose metabolism, glutamate/GABA, glycogen, major depression, PLA_2_

## Abstract

It is firmly believed that the mechanism of action of SSRIs in major depression is to inhibit the serotonin transporter, SERT, and increase extracellular concentration of serotonin. However, this undisputed observation does not prove that SERT inhibition is the mechanism, let alone the only mechanism, by which SSRI’s exert their therapeutic effects. It has recently been demonstrated that 5-HT_2B_ receptor stimulation is needed for the antidepressant effect of fluoxetine *in vivo*. The ability of all five currently used SSRIs to stimulate the 5-HT_2B_ receptor equipotentially in cultured astrocytes has been known for several years, and increasing evidence has shown the importance of astrocytes and astrocyte-neuronal interactions for neuroplasticity and complex brain activity. This paper reviews acute and chronic effects of 5-HT_2B_ receptor stimulation in cultured astrocytes and in astrocytes freshly isolated from brains of mice treated with fluoxetine for 14 days together with effects of anti-depressant therapy on turnover of glutamate and GABA and metabolism of glucose and glycogen. It is suggested that these events are causally related to the mechanism of action of SSRIs and of interest for development of newer antidepressant drugs.

## Introduction: SERT, the 5-HT_2B_ receptor and SSRI

### SSRI targets

It is generally thought that the molecular mechanism of SSRIs is the long-known blockage of serotonin (5-HT) reuptake by its transporter SERT (Fuller and Wong, 1977; Wong and Bymaster, 1995). Binding of SSRIs to this transporter (Langer et al., 1986; Launay et al., 2006; Diaz et al., 2012) leads to elevated extracellular 5-HT levels, assumed to cause their antidepressant effects. SSRIs do exert a direct inhibitory effect on the SERT protein on the neuronal presynaptic plasma membrane (Zhou et al., 2009). Recently the molecular basis for high-affinity recognition of fluoxetine in the SERT molecule was unraveled (Andersen et al., 2014). However, these findings do not prove that SERT inhibition is the mechanism, let alone the only mechanism, by which SSRI’s exert their therapeutic effects in major depression.

Recently Diaz et al. (2012) demonstrated in intact brain a raphe nuclei 5-HT_2B_ receptor with relatively high affinity for SSRIs, *which was indispensable for the antidepressant effect of fluoxetine*. However, SSRI effects exerted via the 5-HT_2B_ receptor had previously been described not only in cultured *neurons* (Launay et al., 2006) but also in cultured *astrocytes* (Kong et al., 2002), where they have been thoroughly studied (Li et al., 2008a, 2009; Zhang et al., 2010). The resultant induction of signaling pathways in glia and neurons has been further characterized in astrocytic and neuronal fractions from mice treated for 2 weeks with fluoxetine. They may play a key role in the antidepressant mechanism of SSRIs. Given the dominant role ascribed to neurons in the mechanisms of SSRIs and other psychiatric drugs it may appear surprising that studies found the major chronic impact of SSRIs in the fluoxetine-treated mice to be on astrocytes. However they are consistent with the growing evidence for a major role of these cells in major depression and its therapy studied in patients (Abdallah et al., 2014a,b), postmortem brain (Rajkowska and Stockmeier, 2013) or in models of this disease (Gosselin et al., 2009; Banasr et al., 2010).

Astrocytes account for ~25% of brain cortical volume and are responsible for at least a corresponding fraction of oxidative energy metabolism, mainly required for maintaining extracellular glutamate, GABA, and K^+^ homeostasis, and synthesis of glutamate and GABA via the glutamine-glutamate (GABA) cycle (reviewed in Hertz, 2011). This cycle is well established in the brain *in vivo*, where it represents the quantitatively most important interaction between neurons and astrocytes. It will be described in more detail below. Astrocytes synthesize all transmitter glutamate and GABA and accumulate most after neuronal release. According to recent research (Duarte and Gruetter, 2013), astrocytes account for an even larger fraction of oxidative brain metabolism, when their role in subsequent *metabolism of released GABA* before its partial return via astrocytes to neurons also is taken into account. These major roles of astrocytes are likely to be relevant for antidepressant effects on glutamate homeostasis, excitatory and inhibitory signaling, and glucose metabolism. Involvement of the glutamine-glutamate (GABA) cycle remains to be studied after SSRI administration, but it has been investigated in patients suffering from major depression (Abdallah et al., 2014a). Moreover, studies of the rapidly acting anti-depressant drugs ketamine or riluzole (which have no known effect on 5-HT_2B_ receptors) have shown that increases in flux in this cycle parallel recovery from experimental and clinical depression (Chowdhury et al., 2008, 2012; Brennan et al., 2010).

Besides discussing the 5-HT_2B_ receptor as an SSRI target, this review will deal with 5-HT_2B_ receptors’ cellular locations; the signaling pathways activated; short term effect on cell signaling; and long-term-effects in cultured astrocytes and in fluoxetine-treated animals. Consequences of long term (14 days) SSRI treatment on gene up-regulation and editing in primary cultures of astrocytes and in neurons and astrocytes freshly isolated from the brains of mice treated with fluoxetine and/or in whole brains from such animals are described in detail. Some of these effects are exerted on genes mediating glutamate/glutamine transport and interconversion and on glutamate and GABA receptor genes. Other effects are exercised on genes which are not directly related to glutamate signaling, but are important for the well-established correlation between recovery from major depression and increase in glucose metabolism in brain (Buchsbaum et al., 1997; Mayberg et al., 2000; Kennedy et al., 2001). Finally, the reviewed studies pinpoint acute and chronic effects on pathways for glycogen turnover. This is important because glycogenolysis is known to impact glutamate formation, learning and longer term neuroplasticity (Gibbs et al., 2007, 2008; Duran et al., 2013). Together, these changes may be the link between the molecular and cellular changes due to 5-HT_2B_ receptor binding and the longer-term impact on depressive symptoms. Selective activation of this receptor or intermediates of its downstream pathways may accordingly constitute potential targets for pharmaceutical development. Such development would be important, since (i) a considerable fraction of patients suffering from major depression do not respond adequately to current antidepressant therapy; (ii) the response is slow except for a few recently tested drugs (O’Leary et al., 2014); and (iii) even the relatively safe SSRIs can have severe side effects if used in pregnant women (Ellfolk and Malm, 2010) or after acute coronary occlusion (Rieckmann et al., 2013).

### The critical importance of the 5-HT_2B_ receptor for SSRI effects

The 5-HT_2B_ receptor was identified in 1987 (Cohen and Fludzinski, 1987) and was thus unknown when SSRIs were introduced and believed to lack relevant receptor effects. Like other 5-HT_2_ receptors, the 5-HT_2B_ receptor is G_q/11_ protein-coupled and stimulates phospholipase C (PLC) to generate diacylglycerol (DAG) and inositol 1,4,5-trisphosphate (IP_3_) by hydrolysis of phosphatidyl-inositol 4,5-bisphosphate (PIP_2_). This triggers IP_3_ receptor-mediated increase of free cytosolic calcium concentration ([Ca^2+^]_i_) (Deecher et al., 1993; Roth et al., 1998; Porter et al., 1999) and additional second messenger effects. During chronic exposure to fluoxetine these second messenger effects may be responsible for the many reported changes in gene expression, as well as alterations in metabolism and the glutamine-glutamate (GABA) cycle that will be described later.

**Figure 1:**
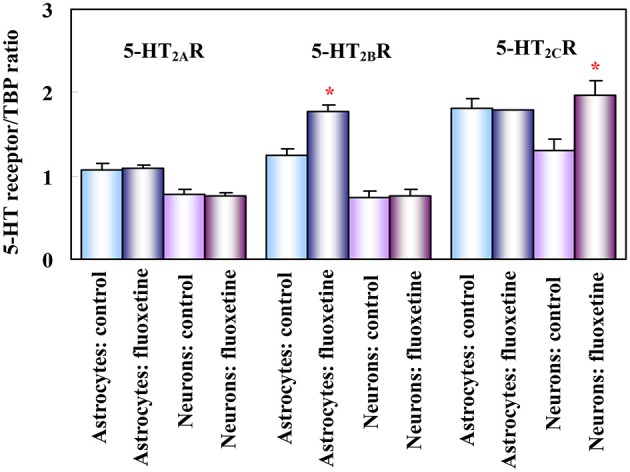
**Effect of fluoxetine on 5-HT_2_ receptor mRNA in astrocytes and neurons obtained separately from both control mice (injected with saline) and mice treated for 14 days with fluoxetine**. mRNA expression was measured by reverse transcription polymerase chain reaction (RT-PCR) of 5-HT_2A_, 5-HT_2B_ and 5-HT_2C_ receptors in astrocytes and neurons isolated by fluorescence-activated cell sorting (FACS) from cerebral hemispheres of two different adult mice strains (FVB/NTg(GFAP-GFP)14Mes/J, providing astrocyte-specific fluorescence, and B6.Cg-Tg(Thy1-YFPH)2Jrs/J, providing neuron-specific fluorescence, although mainly of large glutamatergic neurons). Results are shown for mice chronically treated with fluoxetine (10 mg/kg per day) and for untreated controls. They are means ± SEM of ratios between scanned 5-HT_2A_, 5-HT_2B_ or 5-HT_2C_ receptor expression and scanned expression of TATA-binding protein (TBP), used as housekeeping gene. *n* = 3 (neurons) or 4 (astrocytes). *P > 0.05 vs. control group in astrocytes (5-HT_2B_ receptor) or in neurons (5-HT_2C_ receptor). (Li et al., 2012).

The 5-HT_2B_ receptor is expressed in mouse, rat, and human brain (Kursar et al., 1994; Baez et al., 1995; Bonhaus et al., 1995; Choi and Maroteaux, 1996). In astrocytes obtained from mouse brain (Zhang et al., 2010) its mRNA expression is ~2 times higher than in neurons (Li et al., 2012, see Figure 1). It is also a major 5-HT_2_ receptor in astrocyte cultures (Kong et al., 2002). Neuronal expression has been reported in Purkinje cells (Choi and Maroteaux, 1996) and raphe nuclei (Diaz et al., 2012). Diaz et al. (2012) showed in an *in vivo* rat model that 5-HT_2B_ receptors are needed for long-term behavioral effects of fluoxetine. These effects were abolished in ${\text{5-HT}}_{\text{2B}}^{\text{−/−}}$ mice or after pharmacologic inactivation of 5-HT_2B_ receptors, whereas stimulation by a selective 5-HT_2B_ receptor agonist induced similar responses as fluoxetine in behavioral assays. Fluoxetine-mediated neurogenesis (Manev et al., 2003) was eliminated in 5-HT_2B_ knock-out animals, and acute fluoxetine administration to ${\text{5-HT}}_{\text{2B}}^{\text{−/−}}$ mice induced a much smaller increase in hippocampal 5-HT levels than in wild type mice.

## Acute effects of SSRIs on 5-HT_2B_ receptors and their targets

### Acute effects on 5-HT_2B_ receptors on neurons

5-HT_2B_ receptor effects on SERT or inhibition of 5-HT release has been shown in very young cultures of serotonergic neurons and in neuronal-enriched cultures from the raphe nuclei (Azmitia et al., 1990; Launay et al., 2006). However, these nuclei in addition contain glia cells (Azmitia and Whitaker-Azmitia, 1991), which may also be present in the cultures. Unfortunately our own cultured neurons tolerate fluoxetine poorly. Moreover, they are also young and the immature nervous system is different from the mature brain (e.g., Hertz, 2013). However, future studies to establish potential direct neuronal responses to fluoxetine would be of high importance.

### Glial 5-HT_2B_ binding is critical for *in vivo* effects of SSRIs

Support for the concept that direct binding to 5-HT_2B_ receptors is important for SSRI’s mechanisms of action is that all presently used SSRIs have virtually identical affinity for the 5-HT_2B_ receptor in cultured astrocytes (Zhang et al., 2010). This contrasts the huge differences in their potency as inhibitors of 5-HT uptake (Wong and Bymaster, 1995; Popik, 1999). Also inconsistent with SSRIs owing their therapeutic effects solely to an action on SERT are findings that substantial SERT occupancy (about 80%) occurs at sub-therapeutic SSRI doses, and that increasing doses to clinically effective levels causes only very minor increases in SERT binding (Meyer et al., 2004). SERT-unrelated acute and chronic effects of fluoxetine, including those by 5-HT_2B_ receptor stimulation of astrocytes may therefore be important for their mechanisms of action.

### Acute effect of fluoxetine on 5-HT_2B_ receptors and their targets in cultured astrocytes

Cultured astrocytes are well suited to acute and chronic studies of SSRIs as they can survive undamaged in culture for a long time. Effects on gene expression and editing can be compared with those exerted in the brain *in vivo* after separation of an astrocytic and a neuronal cell fraction as described by Lovatt et al. (2007).

Fluoxetine displaces serotonin binding to cultured astrocytes (Hertz et al., 1979). Kong et al. (2002) found expression of mRNA expression of 5-HT_2B_ and 5-HT_2A_ receptors, but not of 5-HT_2C_ receptors in the cultures (Zhang et al., 1993). Lack of inhibition by ketanserin showed that this was not a 5-HT_2A_ receptor effect. They also obtained quantitative data for displacement of the universal 5-HT_2_ receptor ligand mesulergine by fluoxetine. Based on these data the K_i_ value for fluoxetine’s displacement of 5-HT was calculated to be 70 nM (Hertz et al., 2012). This affinity is 4 times higher than that originally determined for binding of fluoxetine in brain tissue to the 5-HT_2C_ receptor, for which fluoxetine initially was found to have the highest affinity (Wong and Bymaster, 1995).

Studies on post receptor signaling in these glia cells have shown effects on signaling targets *such as growth factors and glycogen synthesis*. Both of these are known to influence neuroplasticity, which is believed to be key factor in the efficacy of SSRIs as antidepressants (Eom and Jope, 2009; Freitas et al., 2013). Acute administration of fluoxetine increases [Ca^2+^]_i_ and stimulates glycogenolysis (a [Ca^2+^]_i_-dependent process) in cultured astrocytes (Zhang et al., 1993; Chen et al., 1995). These effects are not limited to cultured cells, since both fluoxetine and paroxetine support learning in day-old chickens. This effect is inhibited by an antagonist of the 5-HT_2B,C_ receptor and by a glycogenolytic inhibitor (Gibbs and Hertz, 2014). Further evidence that the effects were specific to 5-HT_2B_ binding as opposed to SERT was that the same effects were obtained with almost identical doses of SSRIs (Zhang et al., 2010) that affect 5-HT uptake with widely different potency (Wong and Bymaster, 1995).

**Figure 2:**
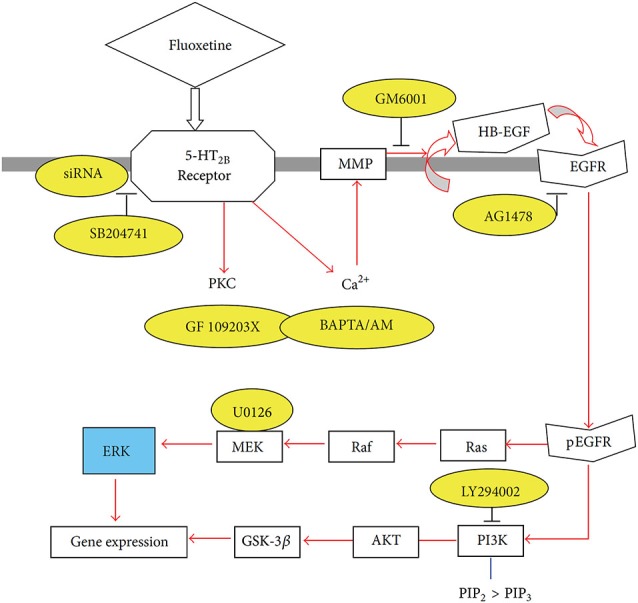
**Schematic illustration of pathways leading to stimulation of extracellular-regulated kinase (ERK) and AKT phosphorylation by fluoxetine in astrocytes**. Fluoxetine binds to 5-HT_2B_ receptors. The activation of the receptors induces protein kinase C (PKC) activity and increase of intracellular Ca^2+^ concentration ([Ca^2+^]_i_) by Ca^2+^ release from intracellular stores. The latter activates Zn-dependent metalloproteinases (MMPs) and leads to shedding of growth factor(s). The released epidermal growth factor receptor (EGFR) ligand stimulates phosphorylation of the EGFR. The downstream target of EGFR, ERK (shown in blue) is phosphorylated via the Ras/Raf/MEK pathway, and AKT is phosphorylated via PI3K pathway. PIK3 is also known to catalyze the formation of PIP_3_ from PIP_2_. During fluoxetine administration, phosphorylation of ERK and AKT was prevented after siRNA administration against the 5-HT_2B_ receptor or after administration of inhibitors (shown in yellow) of this receptor (SB204741), of PKC (GF 109293X), of intracellular Ca^2+^ homeostasis (BAPTA/AM, an intracellular Ca^2+^ chelator), of Zn-dependent MMPs (GM6001), of the receptor-tyrosine kinase of the EGFR (AG1478), of ERK phosphorylation (U0126, a mitogen-activated kinase (MEK) inhibitor) or of the AKT pathway (LY294002, a PI3K inhibitor). This inhibition is an indication of participation of all the inhibited factors in the normal signaling pathway. (Hertz et al., 2012).

The signaling pathway activated by fluoxetine in cultured astrocytes (Li et al., 2008a) is complex (Figure 2). Abolishment of 5-HT_2B_ receptor activity by its siRNA or administration of the 5-HT_2B_ receptor inhibitor SB204741 prevented any response, further validating the concept that the 5-HT_2B_ receptor is directly stimulated. Phosphorylation of extracellular kinases 1 and 2 (ERK_1/2_) was established as an end point without examining further downstream effects, except for a rapid increase in the expression of the immediate early genes cfos and fosB. The increase in [Ca^2+^]_i_ (Chen et al., 1995) leads to activation of metalloproteinases (MMPs) and shedding of growth factor(s), perhaps mainly heparin-binding epidermal growth factor (HB-EGF). This EGF receptor agonist is known to be present in adult brain and to be required for synaptic plasticity (Oyagi et al., 2011).

**Figure 3:**
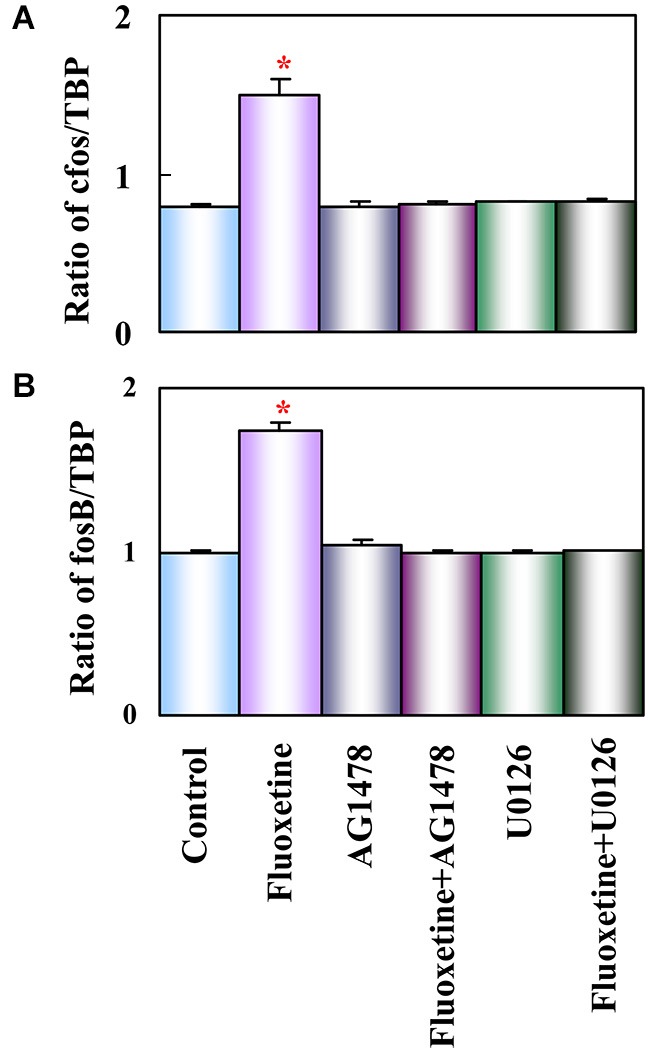
**Activation of EGF receptors and of ERK is required for up-regulation of mRNA expression of c-fos and fosB by fluoxetine in astrocytes**. Cells were incubated for 60 min in serum-free medium in the absence of any drug (Control) or in the presence of 10 μM fluoxetine alone or together with 1 μM AG 1478 or 10 μM U0126. Average mRNA expression (*n* = 3) was quantitated as ratios between c-fos and TBP, used as a house-keeping gene **(A)** and between fosB and TBP **(B)**. SEM values are indicated by vertical bars. *Indicates statistically significant (*P* < 0.05) difference from Control, AG 1478, fluoxetine plus AG 1478, U0126, and fluoxetine plus U0126 groups for c-fos and fosB. (Li et al., 2008a).

Growth factor release may link glial 5-HT_2B_ binding to the longer-term cellular and behavioral changes induced by SSRI stimulation of receptor tyrosine kinases of the epidermal growth factor (EGF) receptor (EGFR). Such an effect of G protein-coupled receptors represents a transactivation process, a common mechanism in astrocytes (Daub et al., 1997; Peavy et al., 2001; Peng, 2004; Du et al., 2009). mRNA expression of the EGFR is approximately 4 times higher in freshly isolated mouse brain astrocytes than in corresponding neurons (Peng et al., 2014). EGFR phosphorylation leads to activation of ERK_1/2_ and of PI3K, with the latter causing AKT phosphorylation (Hertz et al., 2012; Peng et al., 2014). Moreover, released growth factor also acts on neurons (Li et al., 2008b) and may be at least one of the stimuli for the well known fluoxetine-stimulated neurogenesis (Manev et al., 2003) and effects on synaptic activity (Oyagi et al., 2011). Within 1 h the phosphorylation of ERK_1/2_ induces gene expression of cfos and fosB in astrocytes (Li et al., 2008a), shown in Figure 3 to be abolished by inhibitors of the pathway indicated in Figure 2. Fluoxetine also rapidly induces ERK_1/2_-dependent enhancement of gene expression of glial-derived nerve factor (GDNF) in the cultured astrocytes used by Mercier et al. (2004). These cultures differ in several respects from ours, e.g., by being prepared from rats instead of mice and without exposing the cells to the differentiating agent dibutyryl cyclic AMP. It is re-assuring that several aspects of fluoxetine effects are the same. However, some differences were also found, which is not unexpected when cultured cells are used.

A further important step where glial 5-HT_2B_ receptor binding may influence the longer-term cellular and behavioral effects of SSRIs is the impact on glycogen *synthesis*. Glycogen turnover, i.e., interspersed glycogen synthesis and glycogenolysis, is indispensable during learning (Gibbs and Hutchinson, 2012; Hertz et al., 2013a). The acute memory-enhancing, glycogenolysis-dependent effect of both fluoxetine and paroxetine has been mentioned (Gibbs and Hertz, 2014). Knock-out of brain glycogen synthase abolishes learning of new motor and cognitive skills (Duran et al., 2013). It is likely that fluoxetine also affects glycogen synthesis, since the AKT pathway (Figure 2) is stimulated, as shown by AKT phosphorylation in the cultured cells (Hertz et al., 2012; Peng et al., 2014). Phosphorylated AKT in turn inhibits glycogen synthase kinase-3β (GSK-3β) by phosphorylation (Fang et al., 2005). This probably stimulates glycogen synthesis, since activation of glycogen synthase by GSK-3 decreases its activity (Embi et al., 1980; De Sarno et al., 2002).

**Figure 4:**
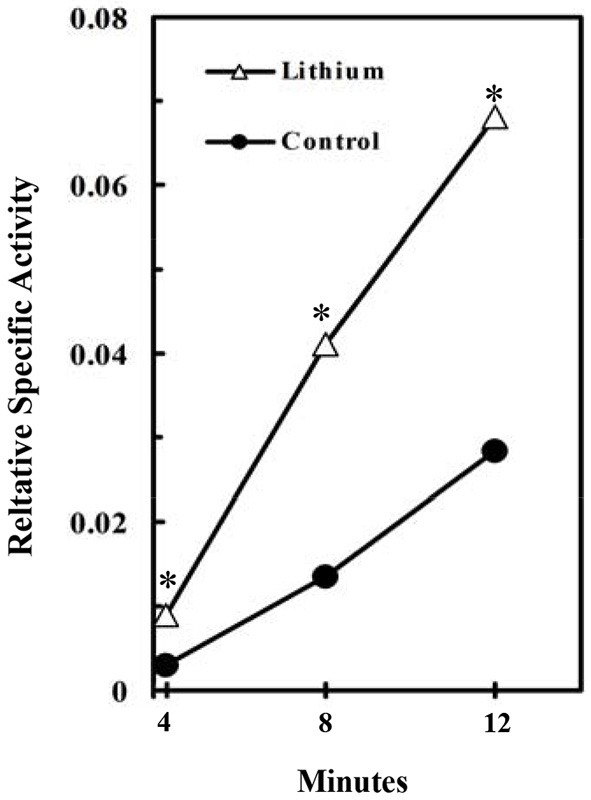
**Stimulation of glycogen synthesis by inhibition of GSK3**. Specific activity of brain glycogen was measured relatively to the specific activity of blood glucose in lithium-treated rats. At 4–12 min after injection of radioactive glucose, 15 μmol LiCl was intracisternally injected into the rats. The brains were frozen 2 h after the injection and incorporation of radioactivity into glycogen determined. *P < 0.05 (4–8 rats at each point). (Plenge, 1976).

Consistent with these findings in cultured astrocytes Plenge (1976) found an acute increase in brain glycogen after administration of Li^+^ (“lithium”), a known inhibitor of GSK3. As shown in Figure 4, this reflected increased glycogen synthesis (Plenge, 1982). Effects of fluoxetine (and of electroconvulsive therapy) by inhibitory phosphorylation of GSK-3 have also been summarized by Gould et al. (2006), and the role of GSK-3 in synaptic plasticity, including memory, discussed by Bradley et al. (2012).

The Jope group found in whole brain that 5-HT_2B_ stimulation or acute fluoxetine administration decreases the levels of phosphorylated GSK3 (Li et al., 2004; Polter et al., 2012). This response was blunted or absent in young mice (Beurel et al., 2012), consistent with astrocytic localization since astrocytes are mainly generated postnatally (Schousboe, 1971; Ge et al., 2012). Other papers from this group showed that deficiency in inhibitory phosphorylation of GSK-3 increases sensitivity to mood disturbances (Polter et al., 2010) and that GSK-3 is required for the antidepressant effect of ketamine (Beurel et al., 2011).

## Further interactions between astrocytes and neurons required for normal function in adult brain

### Astrocytes receive transmitter signals from neurons and release “gliotransmitters” and other neuroactive compounds

The response of astrocytes to the SSRI 5-HT_2B_ agonists is only one example of how astrocyte neurotransmitter receptors may modulate synaptic strength and plasticity. An increasing number of studies have demonstrated that astrocytes express receptors for most neurotransmitters and release neuroactive substances that modulate neuronal activity (Ben Achour and Pascual, 2010). Besides the already mentioned growth factor release in response to stimulation by 5-HT_2B_ receptors (and many other neurotransmitter receptors) they release compounds like adenosine triphosphate (ATP) and glutamate as gliotransmitters. The amount of glutamate released is much smaller than that released from glutamatergic neurons, but it is of special importance because it acts on glutamate receptors which are not located postsynaptically. In this manner astrocytes contribute to regulation of long-term potentiation (LTP), long-term depression (LTD) and neuroplasticity (Ben Achour and Pascual, 2010).

### Astrocytes synthesize all transmitter glutamate and GABA and accumulate most after neuronal release

**Figure 5:**
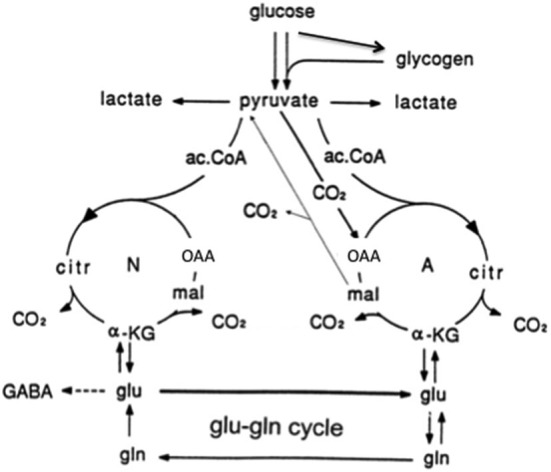
**Cartoon of glucose metabolism via pyruvate in neurons (left—N) and astrocytes (right—A) and of glutamine–glutamate (GABA) cycling**. In both cell types 2 molecules pyruvate are formed from one molecule glucose by glycolysis. Their metabolism via acetyl Coenzyme A (ac.CoA) leads to formation of citrate by condensation with pre-existing oxaloacetate (OAA) in the tricarboxylic acid (TCA), an end-result of the previous turn of the cycle. Citrate oxidation in the TCA cycle includes two decarboxylations, leading to re-formation of OAA, ready for another turn of the cycle, and to production of large amounts of energy (ATP). Pyruvate carboxylation occurs only in astrocytes. It creates a new molecule of OAA, which after condensation with ac.CoA, derived from a second molecule of pyruvate, forms a new molecule of citrate. This process can be used for replacement of worn TCA cycle intermediates. More important in the present context is that α-ketoglutarate (α-KG), one of the intermediates of the TCA cycle can leave the cycle to form glutamate (glu), catalyzed by aspartate aminotransferase and/or glutamate dehydrogenase. In turn, glutamate is amidated to glutamine (gln), catalyzed by the cytosolic and astrocyte-specific enzyme glutamine synthetase. The arrows between neuronal α-KG and glu do not indicate net synthesis but only isotope exchange. After release from astrocytes glutamine is accumulated in glutamatergic and GABAergic neurons (lower line of the glutamine–glutamate(GABA) cycle [Glu–gln cycle]), converted to glutamate (and in GABAergic cells onward to GABA) and released as transmitter. Released glutamate is almost quantitatively re-accumulated in the astrocytic cytosol. Part of the released GABA is also accumulated in astrocytes [upper line of the glutamine–glutamate (GABA) cycle], but its conversion to glutamate requires oxidative metabolism in astrocytes as described in the text. Here, 75–85% of accumulated glutamate is converted to glutamine and re-enters the glutamine–glutamate (GABA) cycle. The remaining 15–25% is oxidatively degraded after re-conversion via α-ketoglutarate to malate, exit of malate to the cytosol, decarboxylation to pyruvate by cytosolic malic enzyme and further pyruvate oxidation in the TCA cycle via ac.CoA. This part must in the long term be replaced by a quantitatively similar de novo production of glutamate from glucose as described above. Alternatively, malate may not exit the mitochondria but after formation of OAA and condensation with ac.CoA be used for re-synthesis of another molecule of glutamate. Under some conditions de novo synthesis of glutamate exceeds its oxidation, leading to an increase in tissue glutamate (e.g., Gibbs et al., 2007; Mangia et al., 2012). Moreover the 15–25% of total flux in the glutamine-glutamate (GABA) cycle, which is resynthesized and oxidized might change, if the equilibrium is disturbed between the activities of enzymes catalyzing the interconversion between glutamate and α-ketoglutarate (aspartate aminotransferase and/or glutamate dehydrogenase) and that catalyzing glutamate conversion to glutamine (glutamine synthetase). This could potentially also happen if the concentrations of the reactants are altered, e.g., as a result of a change in pyruvate carboxylase activity. There is no proof of such effects, but they can also not be excluded. Neuronal re-supply of GABA may be less dependent on the glutamate-glutamine (GABA) cycle than their supply of glutamate, as discussed in the text. (Hertz et al., 2013b).

Neurons cannot carry out glutamate synthesis from glucose because they lack an enzyme (pyruvate carboxylase), which is critical for glutamate synthesis *in vivo* (Shank et al., 1985) and in culture (Yu et al., 1983). The majority of glutamate released as a neurotransmitter is taken up by glial cells. Subsequently glutamate is transferred to neurons in the glutamine-glutamate (GABA) cycle via astrocytic glutamine formation, release of glutamine, and its uptake in neurons, where it is deamidated to glutamate (Hertz and Zielke, 2004; Schousboe et al., 2013). In glutamatergic neurons glutamate is used as a transmitter, and in GABAergic neurons it is converted to GABA (Figure 5). In gray matter (Lebon et al., 2002) of the awake human brain *the rate of this process equals about 75% of the rate of total glucose consumption*, and in the deeply anesthetized, iso-electric brain it is abolished (Sibson et al., 1998; Hyder and Rothman, 2012; Duarte and Gruetter, 2013). The correlation between cycle flux and brain glucose utilization is linear (Sibson et al., 1998; Hyder and Rothman, 2012). Key processes in this massive astrocyte-neuronal interaction are strictly regulated and/or complex. This applies to astrocytic release of glutamine (Nissen-Meyer and Chaudhry, 2013) and neuronal formation of glutamate from glutamine (Palaiologos et al., 1989), which requires concomitant stimulation of glycolysis (Chowdhury et al., 2014; Verkhratsky et al., 2014). This requirement explains part of the linear correlation between glucose utilization and the glutamine-glutamate (GABA) cycle. The cycle also brings previously released transmitter amino acids back to neurons after an initial uptake in astrocytes (Figure 5). This is associated with a small amount of energy expenditure (for glutamate uptake and glutamine formation). Return to astrocytes of previously released transmitter and its transport back to neurons represents the major part of the flux in the glutamine-glutamate (GABA) cycle. Only 15–25% of the flux serves to transfer *newly synthesized glutamate* from astrocytes to neurons (an anaplerotic process) and return glutamate to astrocytes for oxidative degradation (a cataplerotic process). The anaplerotic de novo synthesis requires glucose utilization in astrocytes (one glucose molecule for each molecule of glutamate). Cycle flux is also significantly correlated with glutamate content in human brain (Abdallah et al., 2014a). Since the glutamate content in astrocytes is very low (Lebon et al., 2002), this mainly represents neuronal glutamate.

The GABA component of the glutamine-glutamate (GABA) cycle accounts for ~20% of cycle flux from neurons to astrocytes (Patel et al., 2005; Duarte and Gruetter, 2013). GABA synthesis and metabolism to glutamine shows similarities and differences from production and degradation of glutamate. In contrast to direct astrocytic uptake of most neuronally released glutamate, astrocytically accumulated GABA must first be converted to glutamate in the TCA cycle as described below. This requires condensation of oxaloacetate (OAA) with acetyl Coenzyme A (ac.CoA; Figure 5) but no pyruvate carboxylation (Patel et al., 2005; Duarte and Gruetter, 2013; Lanz et al., 2013). The generated glutamate might be directly converted to GABA. Cultured GABAergic neurons show also a substantial uptake and re-utilization of released GABA (Schousboe et al., 2013). Both of these mechanisms may make GABA synthesis less dependent upon the classical glutamate-glutamine (GABA) cycle. However a recent *in vivo* study in which neuronal reuptake was inhibited showed minimal impact on the fraction of GABA synthesis from glutamine suggesting that direct reuptake may be significantly lower at least in the cerebral cortex (Patel et al., 2015). Further studies will be needed to fully understand the regional and activity dependance of direct neuronal reuptake vs. astrocyte uptake.

The conversion of GABA to glutamate is a complex process, in which GABA initially enters the astrocytic mitochondria, possibly in exchange with glutamate allowing exit of newly synthesized glutamate to the cytosol. It is then transaminated to succinic semialdehyde and oxidized to succinate, which enters the TCA cycle and via oxaloacetate (OAA) and citrate is converted to α-ketoglutarate, from which glutamate is generated by the same transamination that catalyzes the conversion of GABA to succinic semialdehyde. The participation of TCA cycle activity means that complete oxidation also could have occurred after exit of malate (Figure 5). However, Duarte and Gruetter (2013) found no increase in pyruvate carboxylation from previously reported values, indicating no major increase in anaplerosis/cataplerosis, compared to cycling related to glutamatergic signaling.

In the long term, rates of anaplerosis and cataplerosis must be identical (Lebon et al., 2002; Sonnewald, 2014), but this is not necessarly so in the short term, since brain glutamate content can transiently increase. This happens during specific phases of learning (Gibbs et al., 2007), although children with reading difficulties (Pugh et al., 2014) or with ADHD (Carrey et al., 2007) have an increased brain glutamate content. The latter findings might be related to hyperexcitability. In cases of epileptic seizure and even physiological visual stimulation there is an acute increase in brain glutamate (Peca et al., 2010; Mangia et al., 2012). The larger involvement of astrocytic metabolism during anaplerosis/cataplerosis provides increased possibilities for astrocytic regulation of the cycle. Nevertheless even simple astrocytic involvement in return of previously released transmitter provides a possibility for astrocytic regulation, because glutamine can traverse the astrocytic syncytium (Cruz et al., 2007). It cannot even be excluded that incoming glutamate from neuronal release might be re-directed to GABA-ergic neurons and vice-versa.

### K^+^ homeostasis

There is increasing evidence that increased extracellular K^+^ resulting from neuronal excitation is initially accumulated in astrocytes by the astrocytic Na^+^, K^+^-ATPase, which in contrast to the neuronal enzyme is stimulated by above-normal extracellular K^+^ concentrations (reviewed by Hertz et al., 2014b). It is subsequently released via Kir4.1 channels, probably after transport through the astrocytic syncytium, distributing the amount released over a larger area and thus preventing excessive local extracellular K^+^ increase. This allows secondary uptake by the neuronal Na^+^, K^+^-ATPase, preventing neuronal K^+^ depletion. This process is strikingly reminiscent of how astrocytes handle released neuronal glutamate. However in neither case is it known which advantages the energetically costly double uptake provides for the brain.

## Chronic effects of fluoxetine

### Objectives and methodologies

The several weeks delay between SSRI administration and improvement in depressive symptoms show that chronic effects are the most clinically relevant (Nierenberg et al., 2000). The long life span of astrocyte cultures allows chronic studies of both functional properties and gene expression and editing. In the studies described below all effects on gene expression were *confirmed* in isolated neuronal and astrocytic cell fractions (Lovatt et al., 2007) prepared from chronically fluoxetine-treated animals as described in the legend of Figure 1. Our own observations using these methodologies will be supplemented with information from studies by other authors, which provide no information about cellular location(s). While the absence of SERT in cultured astrocytes excludes that the gene effects could be secondary to SERT inhibition, those shown in neurons in intact animals could be SERT-related.

The effects of SSRIs on gene expression and editing in both neurons and astrocytes from fluoxetine-treated animals (10 mg fluoxetine hydrochloride/kg per day for 14 days).are summarized in Table 1. The expression and editing changes in both neurons and astrocytes are present in whole brain as also shown in the Table. Many of the genes studied are relevant for major depression. This includes *a key role for glutamate in major depression and its treatment* (Barbon et al., 2006; Sanacora et al., 2007, 2012; Chowdhury et al., 2008, 2012; Banasr et al., 2010; Hertz et al., 2012; Li et al., 2012; Sanacora and Banasr, 2013; Niciu et al., 2014). *Furthemore some oppositely-directed gene changes have been shown in an animal model of depression, indicating their therapeutic relevance* (Li et al., 2012; Peng et al., 2014).

**Table 1:** **Some genes affected by chronic fluoxetine treatment in different brain preparations**.

Gene	FACS, cerebral astrocytes*	Cultured astrocytes	FACS or otherwise identified cerebral neurons*^+^	Brain (different regions)	Raphe
**ADAR1**	**Unaltered** Li et al. (2012)	**Unaltered** Li et al. (2011a)	**Unaltered** Li et al. (2012)
**ADAR2**	**Up** Li et al. (2012)	**Up** Li et al. (2011a)	**Unaltered** Li et al. (2012)	**Up** Li et al. (2011a)
**GluK1**	**Absent** Li et al. (2012)	**Absent** Li et al. (2011a)	Absent** Li et al. (2012)
**GluK2**	**Up** Li et al. (2012)	**Up** Li et al. (2011a)	**Unaltered** Li et al. (2012)	**Up** Li et al. (2011a)
**GluK2 editing**	**Up** Li et al. (2012)	**Up** Li et al. (2011a)	**Unaltered** Li et al. (2012)	**Up** Li et al. (2011a)
**GluK3**	**Unaltered** Li et al. (2012)	**Unaltered** Li et al. (2011a)	**Unaltered** Li et al. (2012)
**GluK4**	**Unaltered** Li et al. (2012)	**Unaltered** Li et al. (2011a)	**Up** Li et al. (2012)
**GluA1**				**Up** Barbon et al. (2011)
**GluA2**			**Up in dendrites** Rubio et al. (2013)	**Up** Ampuero et al. (2010), Vialou et al. (2010), Barbon et al. (2011)
**GluA3**				**Up** Barbon et al. (2011)
**GluA4**				**Up** Barbon et al. (2011)
**mGlu5**	**Unaltered** Hertz et al. (2014a)		**Unaltered** Hertz et al. (2014a)
**mGlu7**				**Up** Ampuero et al. (2010), O’Connor et al. (2013)
**cPLA_2a_**	**Up** Li et al. (2012)	**Up** Li et al. (2009)	**Unaltered** Li et al. (2012)	**Up** Rao et al. (2006)
**sPLA_2_**	**Unaltered** Li et al. (2012)	**Unaltered** Li et al. (2009)	**Up** Li et al. (2012)	**Up** Peng et al. (2014)
**iPLA_2_**	**Unaltered** Li et al. (2012)	**Unaltered** Li et al. (2009)	**Unaltered** Li et al. (2012)
**Ca_v_1.2**	**Up** Du et al. (2014)	**Up** Du et al. (2014)	**Unaltered** Du et al. (2014)
**Ca_v_1.3**	**Unaltered** Du et al. (2014)		**Unaltered** Du et al. (2014)
**5-HT_2A_ receptor**	**Unaltered** Li et al. (2012)		**Unaltered** Li et al. (2012)		
**5-HT_2B_ receptor**	**Up** Li et al. (2012)	**Up** Hertz et al. (2014a)	**Unaltered** Li et al. (2012)		
**5-HT_2B_ editing**	**Up** Li et al. (2012)	**Up** Hertz et al. (2014a)	**Unaltered** Li et al. (2012)		
**5-HT_2C_ receptor**	**Unaltered** Li et al. (2012)	**Unaltered** Hertz et al. (2014a)	**Up** Li et al. (2012)		
**5-HT_2C_ editing**				**Up** Gurevich et al. (2002), Englander et al. (2005), Schmauss et al. (2010)	
**5-HT_1A_ receptor**	**Unaltered** Peng et al. (2014)		**Unaltered** Peng et al. (2014)	**Unaltered** Le Poul et al. (2000), Johnson et al. (2009)	**Down** Le Poul et al. (2000)
**5-HT_1B_ receptor**				**Up or Unaltered** Anthony et al. (2000), Le Poul et al. (2000)	**Down** Anthony et al. (2000)
**SERT**	**Absent** Li et al. (2012)	**Absent** Kong et al. (2002)	**Absent** Li et al. (2012)	**Unaltered** Johnson et al. (2009)	**Down** Lesch et al. (1993)
**EGF receptor**	**Unaltered** Peng et al. (2014)		**Unaltered** Peng et al. (2014)		
**Nucleoside transporter ENT2**	**Up** Li et al. (2013)		**Up** Li et al. (2013)		

*The cells isolated by FACS were freshly obtained (see text) from mice treated for 2 weeks with fluoxetine hydrochloride, 10 mg/kg per day, i.p. The cultured cells were treated with fluoxetine concentrations between 1 and 10 μM. for 14 days. For brain and raphe the treatment varied between 7 and 14 days (e., 14 days for sPLA_2_, but 7 days for GluK2). ^+^FACS unless otherwise indicated. **present at low density in cultured hippocampal neurons (Li et al., 2011a).

### Effects on glutamate, GABA and energy metabolism

Patients suffering from major depression show evidence of increased glutamatergic activity (Mitani et al., 2006; Hashimoto et al., 2007; Kanner, 2014) and decreased GABA-ergic activity (Bajbouj et al., 2006; Kanner, 2014) as well as of cortical GABA levels (Sanacora et al., 2004). Successful therapy of major depression lowers cortical glutamate in brain (Abdallah et al., 2014a) and raises GABA (Sanacora et al., 2002; Bhagwagar et al., 2004).

Glutamine synthetase is down-regulated in brains from depressed patients (Rajkowska and Stockmeier, 2013). This is consistent with the increased amino acid neurotransmitter (glutamate and GABA) cycling between astrocytes and neurons after antidepressant treatment with ketamine or riluzole (Chowdhury et al., 2008, 2012), Moreover, inhibition of glutamine synthetase or of the transporter mediating glutamine uptake into neurons causes depression-like symptoms in animal models (Lee et al., 2013).

In a recent ^13^C MRS study no significant difference in cycling flux, determined from glutamate and glutamine ^13^C labeling from [1-^13^C]glucose, (0.19 ± 0.05 (SD) vs. 0.18 ± 0.04 (SD) μmol/g per min) was found between healthy and depressed individuals (Abdallah et al., 2014a). The rate of GABA synthesis was also unaltered. However, inhibition of glutamine synthase or glutamate uptake does not necessarily decrease glutamate flux to glutamine. The flux can still be maintained if glutamate release is not inhibited but at the cost of higher extracellular and glial glutamate levels, as has been seen when glial glutamate uptake or glutamine synthase is inhibited in animal models (Rothstein et al., 1996; Eid et al., 2012). The elevated glutamate would be expected to alter synaptic function. Furthermore it could lead to a change in the ratio of released glutamate that is oxidized in astrocytes vs. glutamate that is directly converted to glutamine.

In patients suffering from depression, brain glucose metabolism is reduced (Little et al., 1996, 2005; Videbech, 2000; Rasgon et al., 2008; Abdallah et al., 2014a) in parallel with the severity of the illness (Kimbrell et al., 2002). Normalization occurs following SSRI treatment (Buchsbaum et al., 1997; Mayberg et al., 2000; Kennedy et al., 2001). Anti-depressant doses of ketamine increase oxidative metabolism in neuronal and glial cells in the brain *in vivo* (Chowdhury et al., 2012). Consistent with this therapeutic effect Abdallah et al. (2014a) found a large (one quarter) decrease in the rate of glucose oxidation by glutamatergic neurons in depressed patients.

On account of the linear relationship between neuronal activity and neuronal oxidative demand beyond isoelectricity, the metabolic decrease in glutamatergic neurons suggests a drastic reduction of neuronal activity. An increased cycling after treatment with riluzole or ketamine might remedy this deficiency regardless whether or not decreased glutamate cycling is a key component of the pathophysiology of major depression. Increased energy metabolism in glutamatergic neurons is required not only for release of neurotransmitter but probably even more for effects exerted via their glutamate receptors.

In conclusion, in spite of convincing evidence for alterations in glutamine-glutamate (GABA) fluxes and associated energetics in animal models of major depression and its treatment there are still unanswered questions in human depression. This is perhaps partly because the cycle is of key importance for both glutamatergic and GABAergic signaling, and partly because there are many different glutamate receptors. Effects of chronic fluoxetine treatment of mice on glutamate and GABA receptors in whole brain, astrocytes and or neurons might provide further clues.

### Glutamate and GABA receptor and transporter genes

Fluoxetine effects on up-regulation and/or editing of genes of several glutamate receptors are shown in Table 1. Receptor up-regulation might potentially correct the abnormal ratio between energetics and the glutamine-glutamate (GABA) cycle observed by Abdallah et al. (2014a), whereas the effects of editing may vary between receptors and editing sites. Editing requires ADARs, a family of adenosine deaminases, which catalyze deamination of adenosine to inosine in mRNAs. This changes the amino acids in the translated protein sequence, since inosine is read as guanosine (Bass, 2002). ADAR 2 is upregulated in astrocytes but not in neurons in mice treated with fluoxetine (Table 1). GluK2 is up-regulated and edited in astrocytes at all its 3 editing sites (Li et al., 2011a, 2012), and GluK4 is upregulated in neurons (Li et al., 2012). The Gluk2 editing may be the reason why a normal increase in [Ca^2+^]_i_ in cultured astrocytes in response to 100 μM glutamate is abolished by fluoxetine treatment (Li et al., 2011a). The human GluK2c splice variant in brain is mainly expressed in non-neuronal cells (Barbon et al., 2008). Mice with GluK2 receptor knock-out, exhibit less anxious or more risk-taking type behavior and less manifestation of despair (Shaltiel et al., 2008).

Obsessive-compulsive disorder (OCD) is genetically linked to abnormalities in the GluK2 gene, Grik2 (Delorme et al., 2004; Sampaio et al., 2011). The neuronal up-regulation of GluK4 by fluoxetine may appear paradoxical since genetic ablation of this receptor subunit causes anxiolytic and antidepressant-like behavior in mice (Catches et al., 2012). However, there is a risk of elevated suicidality during initiation of antidepressant therapy (Fava and Rosenbaum, 1991; Kraus et al., 2010; Trivedi et al., 2011; Singh et al., 2013), which might be related to this up-regulation.

The GluA2 gene is expressed in both neurons and astrocytes (Cahoy et al., 2008), and it is up-regulated in whole brain by fluoxetine treatment (Ampuero et al., 2010; Vialou et al., 2010; Barbon et al., 2011). Up-regulation in neuronal dendrites leads to structural plasticity (Rubio et al., 2013). Mice susceptible to chronic social defeat show a significant decrease in GluA2 levels, while resilient mice showed increased GluA2 levels (Vialou et al., 2010). GluA1 (Barbon et al., 2006; Ampuero et al., 2010) and GluA4 (Barbon et al., 2011) are also upregulated in brains of fluoxetine-treated animals, but GluA3 is less affected (Barbon et al., 2006). In contrast to the lack of fluoxetine effect on mGlu5 in both astrocytes and neurons (Table 1) and of an unaltered mGluR2 and mGluR3 expression in whole brain in major depression (Muguruza et al., 2014), mGluR7 is up-regulated in whole brain after fluoxetine treatment (Ampuero et al., 2010; O’Connor et al., 2013). Very little information is available about possible fluoxetine effect on NMDA receptors. The GABA-synthesizing enzyme glutamic acid decarboxylase (GAD) is upregulated in hippocampus but not in prefontal cortex by 2 weeks of fluoxetine treatment (Guirado et al., 2012). A hippocampal up-regulation of the GABA_B_ receptor has also been reported (Sands et al., 2004). A decrease in cerebrospinal fluid of patients with depression of the neurosteroid 3α-hydroxy 5α-pregnan-20-one (allopregnanolone), which enhances GABA action at GABA_A_ receptors, is corrected by fluoxetine treatment (Pinna et al., 2009). The likely reason for this is that fluoxetine, sertraline, and paroxetine acutely cause a large decrease of the K_m_ for conversion of 5α-dihydroprogesterone to allopregnanolone by human 3α-HSD type III (Griffin and Mellon, 1999). In turn, 5α-dihydroprogesterone is formed from progesterone (and some other substrates) by the rate-limiting enzyme 5α-reductase. In cerebellum this enzyme is mainly expressed in astrocytes and oligodendrocytes (Kiyokage et al., 2014). However, in frontal cortex, where allopregnanolone is down-regulated in depressed patients, it was reported to be synthesized in glutamatergic neurons (Agis-Balboa et al., 2014). A decrease of corticolimbic allopregnanolone content induced in mice by social isolation as well as the induced behavioral changes are sterospecifically normalized by fluoxetine by a mechanism independent from 5-HT reuptake inhibition (Pinna et al., 2009). *Thus, fluoxetine facilitates GABA_A_ receptor neurotransmission and effectively ameliorates depression by stimulating brain steroidogenesis in a SERT-independent manner*.

### Phospholipase genes

A polymorphism in the calcium-dependent phospholipase 2 (cPLA_2_) gene is connected with increased risk for major depression (Pae et al., 2004). This enzyme is strongly expressed in astrocytes (Lautens et al., 1998; Balboa and Balsinde, 2002; Sun et al., 2004). In cultured astrocytes cPLA_2_ causes transactivation of the EGF receptor and ERK_1/2_ phosphorylation (Xia and Zhu, 2011). Its activation releases the unsaturated fatty acid arachidonic acid from membrane-bound phospholipids (Felder et al., 1990; Qu et al., 2003; Rapoport, 2008). In agreement with an up-regulation reported in whole brain (Rapoport, 2008), Li et al. (2009) showed a slow and selective up-regulation of mRNA and protein expression of cPLA_2a_, the major cPLA_2_ isoform, in cultured mouse astrocytes during chronic incubation with fluoxetine (Table 1). The up-regulation was abrogated by the 5-HT_2B_ antagonist SB 204741, and by inhibitors of the fluoxetine signaling pathway shown in Figure 2. Up-regulation, specifically of cPLA_2a_ was confirmed in freshly dissociated mouse astrocytes after 2 weeks fluoxetine treatment, whereas no effect was found in neurons (Li et al., 2012).

sPLA_2_ is upregulated by fluoxetine in neurons and whole brain, but not in astrocytes (Table 1). Some subtypes of sPLA_2_ also stimulate arachidonic acid release (Murakami et al., 1998). Exogenously applied sPLA_2_ causes an increase in neurotransmitter release from cultured hippocampal neurons (Wei et al., 2003).

**Figure 6:**
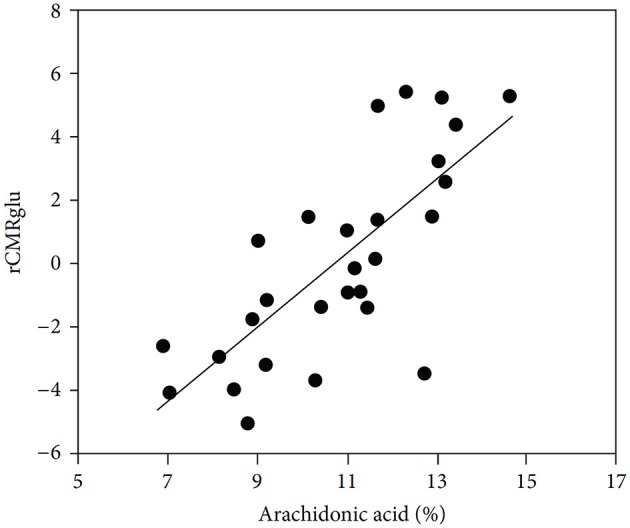
**Correlation between cerebral metabolic rate of glucose metabolism and plasma arachidonic acid levels**. Correlation between arachidonic acid as a percentage of total phospholipid polyunsaturated essential fatty acids and glucose utilization (rCMRglu) measured by fluoro-deoxyglucose PET scan in an area of right temporoparietal cortex that included precentral gyrus, superior temporal gyrus, and inferior parietal lobule in medication-free patients. (Sublette et al., 2009).

*One of the many effects of arachidonic acid is to stimulate glucose metabolism in cultured astrocytes* (Yu et al., 1993). So does treatment with 10 μM fluoxetine for 24 h (Allaman et al., 2011), which might have sufficed to induce an increase in cPLA_2_, whereas acute exposure to fluoxetine has no corresponding effect (L. Peng and L. Hertz, unpublished experiments). *Stimulation of glucose metabolism by arachidonic acid in vivo* (Sublette et al., 2009) may be important in the treatment of depressive illness, as shown in Figure 6. Arachidonic acid in addition stimulates glycogenolysis (Sorg et al., 1995; Hertz et al., 2015) and seems to enhance signaling via glutamatergic receptors of the AMPA and mGluR subtypes (Schaeffer and Gattaz, 2008).

Brain glycogenolysis is activated by a multitude of transmitters (Xu et al., 2014; Hertz et al., 2015), Increased glycogenolysis has been found in paroxetine-treated mice (Webhofer et al., 2013) and in astrocyte cultures treated with 10 μM fluoxetine for more than 1 week, whereas shorter treatment led to a decrease (Kong et al., 2002). Astrocytic glutamate production depends on glycogenolysis (Gibbs et al., 2007). However, glycogenolysis has additional wide-reaching effects on neuroplastic changes, which are likely to be associated with chronic actions of SSRIs. Thus, Duran et al. (2013) found an absence of appropriate postsynaptic LTP responses to high-frequency stimulation in alert, behaving mice with a specific knock-out of brain glycogen synthase.

The acute effect of fluoxetine on GSK-3 via PI3K/AKT and the demonstration that the GSK-3 inhibitor lithium stimulates glycogen synthesis (Plenge, 1982) suggest that *potential chronic effects of fluoxetine on glycogen formation might be very important for its mechanism of action*. This conclusion is consistent with the suggestion by Li and Jope (2010) that GSK-3 is a central modulator of mood regulation. Recent studies by Gu and Peng showed a tripling of astrocytic glycogen content after 14 days of treatment with 1 μM fluoxetine, but a caveat is that with this concentration of fluoxetine the transient decrease in glycogenolysis described above (Kong et al., 2002) may not have subsided after 2 weeks. However, the findings by Plenge (1982) of acute effects of lithium strongly suggest that the stimulation of the PI3K/AKT pathway by chronic treatment with 10 μM fluoxetine does stimulate glycogen synthesis, and treatment of cultured astrocytes with 10 μM fluoxetine treatment for 14 days increased fluoxetine-induced AKT phosphorylation (Hertz et al., 2012). In contrast, chronic lithium treatment abolishes the increased incorporation of glucose into glycogen seen after acute exposure to lithium (Plenge, 1982). *The importance of SSRI-induced effects on glycogen is consistent with its crucial involvement in astrocytic signaling* (Gibbs et al., 2007; Obel et al., 2012; Hertz et al., 2013a, 2014b; Mangia et al., 2013; Xu et al., 2013; Webhofer et al., 2013).

### The calcium channel gene Ca_v_1.2

[Ca^2+^]_i_ is low in resting cells (~100 nM, compared to 1–2 mM in the extracellular fluid). *Increases in [Ca^2+^]_i_ are an essential component of virtually all astrocytic activities* (e.g., formation of glutamate, glycogenolysis, and effects of many transmitters) (Gibbs et al., 2007; Hertz et al., 2015). [Ca^2+^]_i_ increase is also required for transmitter-induced stimulation of several TCA-cycle enzymes and glutaminase (Halestrap, 1989; Denton and McCormack, 1990; Rutter et al., 1996) and for a direct stimulation of oxidative phosphorylation (Gaspers and Thomas, 2008) in many cell types. Such enzyme stimulations have been demonstrated in cultured astrocytes (Subbarao and Hertz, 1991; Huang and Hertz, 1995; Huang et al., 2000; Hertz et al., 2010). Ca^2+^ transport across the cell membrane is therefore of great importance for astrocytic functions. One route of entry is via L-channels opened by membrane depolarization (Li et al., 2011b; Du et al., 2014). The evoked [Ca^2+^]_i_ rise is increased after chronic treatment with fluoxetine, because of up-regulation of the L-channel gene Ca_v_1.2 (Du et al., 2014), shown both in cultured cells and in astrocytes freshly obtained from fluoxetine-treated mice. It is not up-regulated in neurons (Table 1). The fluoxetine-induced up-regulation counteracts a down-regulation of capacitative Ca^2+^ uptake via store-operated channels (Socs), found in astrocytes after treatment with fluoxetine (Li et al., 2011b). The antibipolar drug carbamazepine has a similar effect on Socs in both astrocytes (Yan et al., 2013) and neurons (unpublished), but it has no effect on Ca_v_1.2. A similar neuronal down-regulation of Socs by fluoxetine would be advantageous by reducing the ability of stress-released neuropeptide corticotropin-releasing hormone (CRH) to decrease dendritic spine density in an NMDA- and Ca^2+^-dependent process (Andres et al., 2013). SSRI effects on [Ca^2+^]_i_ is not limited to cultured cells, since citalopram or fluoxetine acutely induces astrocytic [Ca^2+^]_i_ increase in mouse brain slices (Schipke et al., 2011).

### 5-HT receptors genes and SERT

Fluoxetine interactions with 5-HT receptors are age-dependent (Sarkar et al., 2014) and only effects on adult individuals will be discussed. In contrast to the 5-HT_2B_ receptor’s up-regulation by 14 days of *in vivo* treatment with fluoxetine, the astrocytic 5-HT_2A_ and 5-HT_2C_ receptors are unaltered (Table 1). After 2 weeks fluoxetine treatment all editing sites in the 5-HT_2B_ receptor become edited in astrocytes (Li et al., 2012). In cultured astrocytes up-regulation of the 5-HT_2B_ receptor occurs slowly (Hertz et al., 2014a). Editing of the receptor is faster and *abolishe*s the 5-HT-induced increase in IP_3_ turnover. This together with a similar up-regulation and editing of 5-HT_2C_ receptors in neurons (Table 1) are consistent with an early suggestion that chronic SSRI treatment reduces 5-HT_2C_/5-HT_2B_ receptor responsivity in rats (Lightowler et al., 1994).

In whole brain fluoxetine-induced editing of the HT_2C_ receptor has repeatedly been described (Niswender et al., 2001; Gurevich et al., 2002; Englander et al., 2005). It requires PKC and ADAR activity (Schmauss et al., 2010). The fluoxetine-induced changes in editing are opposite to those seen in suicide victims (Gurevich et al., 2002). These findings might suggest that a reduced agonist activity after editing (see above) neutralizes a reported anxiogenic effect of the un-edited 5-HT_2C_ receptor (Kennett et al., 1997; Vicente and Zangrossi, 2014). It is consistent with this conclusion that Vicente and Zangrossi (2014) found acute injection of the 5-HT_2C_ receptor agonist MK-212 into the amygdala to have anxiogenic effect, and that chronic treatment with fluoxetine abolished the anxiety. However, there is also strong evidence that acute administration of *specific* agonists of 5-HT_2C_ receptor are therapeutically effective in depression, obsessive compulsive disorder and anxiety (Jenck et al., 1998; Martin et al., 1998; Leysen, 1999; Cryan and Lucki, 2000; Rosenzweig-Lipson et al., 2007).

The expression of the inhibitory 5-HT_1A_ autoreceptor, suggested to contribute to the slow manifestation of the therapeutic effect of SSRIs (Blier and De Montigny, 1983), was unaltered after fluoxetine treatment in both neurons and astrocytes (Peng et al., 2014) and whole brain (Le Poul et al., 2000; Johnson et al., 2009). This does not mean that its activity could not have decreased, since a fluoxetine-induced internalization of the receptor was shown by Descarries and Riad (2012) and a reduced 5-HT_1A_ receptor expression was found in the raphe nuclei (Le Poul et al., 2000).

The cell culture finding that SERT is absent in astrocytes (Kong et al., 2002) was confirmed in freshly isolated astrocytes from the cerebral hemispheres, excluding raphe nuclei (Peng et al., 2014). This also applies to neurons, although a minor presence cannot be excluded (Table 1). Expression of the EGF receptor, involved in 5-HT_2B_ receptor signaling (Figure 2), is not altered by chronic fluoxetine treatment in either astrocytes or neurons (Peng et al., 2014). However mRNA of the equilibrative nucleoside transporter ENT2 is increased in neurons and even more in astrocytes after fluoxetine treatment (Table 1), probably a reflection of the fluoxetine-induced increase in cell generation (Manev et al., 2003).

The 5-HT_2B_ receptor is downregulated in mice becoming anhedonic after chronic stress (Li et al., 2012). No similar downregulation was found of the 5-HT_2C_ receptor, but anhedonia is only one component of depressive symptomatology, and effects on the 5-HT_2C_ receptor may mainly influence anxiety.

## Conclusions

The importance of the 5-HT_2B_ receptor in the mechanism of action of SSRIs has been demonstrated both *in vivo* (Diaz et al., 2012) and in cultured astrocytes (Li et al., 2008a). This paper elucidates its pathway, emphasizes astrocytic-neuronal interactions in brain function and shows identical effects of fluoxetine on astrocytic gene expression in our cultured astrocytes, *expressing no SERT*, and in drug treated animals. The latter study also found neuronal effects. The signaling pathway, studies by other authors and the observed gene effects point towards the importance in SSRI’s mechanism of action of glucose and glycogen metabolism, glutamate and GABA turnover and signaling, cPLA_2_ and sPLA_2_ activities, arachidonic acid, cellular Ca^2+^ regulation, and alterations in 5-HT_2_ receptor expression and editing. Many of the observed effects might be useful targets for drug development.

## Contribution statement

Professors Hertz and Rothman wrote and revised the manuscript. Professor Rothman is also an author of many of the studies referred to. Professor Peng approved and corrected the initially submitted manuscript, had designed the many experiments from her own group (partly with some input from Professor Hertz) and supported all research from her group. Dr. Li provided essential help to Professor Hertz by preparing the reference list and and quite complex Table 1. She also performed the majority of the experiments in Professor Peng’s group related to this review.

## Conflict of interest statement

The authors declare that the research was conducted in the absence of any commercial or financial relationships that could be construed as a potential conflict of interest.

## References

[B1] Abdallah C. G. Jiang L. De Feyter H. M. Fasula M. Krystal J. H. Rothman D. L.. (2014a). Glutamate metabolism in major depressive disorder. Am. J. Psychiatry [Epub ahead of print]. 10.1176/appi.ajp.2014.14010067 25073688 PMC4472484

[B2] Abdallah C. G. Niciu M. J. Fenton L. R. Fasula M. K. Jiang L. Black A.. (2014b). Decreased occipital cortical glutamate levels in response to successful cognitive-behavioral therapy and pharmacotherapy for major depressive disorder. Psychother. Psychosom. 83, 298–307. 10.1159/000361078 25116726 PMC4164203

[B3] Agis-Balboa R. C. Guidotti A. Pinna G. (2014). 5α-reductase type I expression is downregulated in the prefrontal cortex/Brodmann’s area 9 (BA9) of depressed patients. Psychopharmacology (Berl) 231, 3569–3580. 10.1007/s00213-014-3567-5 24781515 PMC6223254

[B4] Allaman I. Fiumelli H. Magistretti P. J. Martin J. L. (2011). Fluoxetine regulates the expression of neurotrophic/growth factors and glucose metabolism in astrocytes. Psychopharmacology (Berl) 216, 75–84. 10.1007/s00213-011-2190-y 21301813

[B5] Ampuero E. Rubio F. J. Falcon R. Sandoval M. Diaz-Veliz G. Gonzalez R. E.. (2010). Chronic fluoxetine treatment induces structural plasticity and selective changes in glutamate receptor subunits in the rat cerebral cortex. Neuroscience 169, 98–108. 10.1016/j.neuroscience.2010.04.035 20417256

[B6] Andersen J. Stuhr-Hansen N. Zachariassen L. G. Koldsø H. Schiøtt B. Strømgaard K.. (2014). Molecular basis for selective serotonin reuptake inhibition by the antidepressant agent fluoxetine (Prozac). Mol. Pharmacol. 85, 703–714. 10.1124/mol.113.091249 24516100

[B7] Andres A. L. Regev L. Phi L. Seese R. R. Chen Y. Gall C. M.. (2013). NMDA receptor activation and calpain contribute to disruption of dendritic spines by the stress neuropeptide CRH. J. Neurosci. 33, 16945–16960. 10.1523/JNEUROSCI.1445-13.2013 24155300 PMC3807024

[B8] Anthony J. P. Sexton T. J. Neumaier J. F. (2000). Antidepressant-induced regulation of 5-HT(1b) mRNA in rat dorsal raphe nucleus reverses rapidly after drug discontinuation. J. Neurosci. Res. 61, 82–87. 10.1002/1097-4547(20000701)61:1<82::aid-jnr10>3.0.co;2-e 10861803

[B9] Azmitia E. C. Murphy R. B. Whitaker-Azmitia P. M. (1990). MDMA (ecstasy) effects on cultured serotonergic neurons: evidence for Ca2(+)-dependent toxicity linked to release. Brain Res. 510, 97–103. 10.1016/0006-8993(90)90732-q 1969761

[B10] Azmitia E. C. Whitaker-Azmitia P. M. (1991). Awakening the sleeping giant: anatomy and plasticity of the brain serotonergic system. J. Clin. Psychiatry 52(Suppl.), 4–16. 1752858

[B11] Baez M. Kursar J. D. Helton L. A. Wainscott D. B. Nelson D. L. (1995). Molecular biology of serotonin receptors. Obes. Res. 3, 441S–447S 10.1002/j.1550-8528.1995.tb00211.x 8697042

[B12] Bajbouj M. Lisanby S. H. Lang U. E. Danker-Hopfe H. Heuser I. Neu P. (2006). Evidence for impaired cortical inhibition in patients with unipolar major depression. Biol. Psychiatry 59, 395–400. 10.1016/j.biopsych.2005.07.036 16197927

[B13] Balboa M. A. Balsinde J. (2002). Involvement of calcium-independent phospholipase A2 in hydrogen peroxide-induced accumulation of free fatty acids in human U937 cells. J. Biol. Chem. 277, 40384–40389. 10.1074/jbc.m206155200 12181317

[B14] Banasr M. Chowdhury G. M. Terwilliger R. Newton S. S. Duman R. S. Behar K. L.. (2010). Glial pathology in an animal model of depression: reversal of stress-induced cellular, metabolic and behavioral deficits by the glutamate-modulating drug riluzole. Mol. Psychiatry 15, 501–511. 10.1038/mp.2008.106 18825147 PMC3347761

[B15] Barbon A. Caracciolo L. Orlandi C. Musazzi L. Mallei A. La Via L.. (2011). Chronic antidepressant treatments induce a time-dependent up-regulation of AMPA receptor subunit protein levels. Neurochem. Int. 59, 896–905. 10.1016/j.neuint.2011.07.013 21839792

[B16] Barbon A. Gervasoni A. LaVia L. Orlandi C. Jaskolski F. Perrais D.. (2008). Human GluR6c, a functional splicing variants of GluR6, is mainly expressed in non-nervous cells. Neurosci. Lett. 434, 77–82. 10.1016/j.neulet.2008.01.049 18289788

[B17] Barbon A. Popoli M. La Via L. Moraschi S. Vallini I. Tardito D.. (2006). Regulation of editing and expression of glutamate alpha-amino-propionic-acid (AMPA)/kainate receptors by antidepressant drugs. Biol. Psychiatry 59, 713–720. 10.1016/j.biopsych.2005.10.018 16460696

[B18] Bass B. L. (2002). RNA editing by adenosine deaminases that act on RNA. Annu. Rev. Biochem. 71, 817–846. 10.1146/annurev.biochem.71.110601.135501 12045112 PMC1823043

[B19] Ben Achour S. Pascual O. (2010). Glia: the many ways to modulate synaptic plasticity. Neurochem. Int. 57, 440–445. 10.1016/j.neuint.2010.02.013 20193723

[B20] Beurel E. Mines M. A. Song L. Jope R. S. (2012). Glycogen synthase kinase-3 levels and phosphorylation undergo large fluctuations in mouse brain during development. Bipolar Disord. 14, 822–830. 10.1111/bdi.12023 23167932 PMC3505070

[B21] Beurel E. Song L. Jope R. S. (2011). Inhibition of glycogen synthase kinase-3 is necessary for the rapid antidepressant effect of ketamine in mice. Mol. Psychiatry 16, 1068–1070. 10.1038/mp.2011.47 21502951 PMC3200424

[B22] Bhagwagar Z. Wylezinska M. Taylor M. Jezzard P. Matthews P. M. Cowen P. J. (2004). Increased brain GABA concentrations following acute administration of selective serotinonin reuptake inhibitor. Am. J. Psychiatry 161, 368–370. 10.1176/appi.ajp.161.2.368 14754790

[B23] Blier P. De Montigny C. (1983). Electrophysiological investigations on the effect of repeated zimelidine administration on serotonergic neurotransmission in the rat. J. Neurosci. 3, 1270–1278. 6304261 10.1523/JNEUROSCI.03-06-01270.1983 PMC6564602

[B24] Bonhaus D. W. Bach C. DeSouza A. Salazar F. H. Matsuoka B. D. Zuppan P.. (1995). The pharmacology and distribution of human 5-hydroxytryptamine2B (5-HT_2B_) receptor gene products: comparison with 5-HT_2A_ and 5-HT_2C_ receptors. Br. J. Pharmacol. 115, 622–628. 10.1111/j.1476-5381.1995.tb14977.x 7582481 PMC1908489

[B25] Bradley C. A. Peineau S. Taghibiglou C. Nicolas C. S. Whitcomb D. J. Bortolotto Z. A.. (2012). A pivotal role of GSK-3 in synaptic plasticity. Front. Mol. Neurosci. 5:13. 10.3389/fnmol.2012.00013 22363262 PMC3279748

[B26] Brennan B. P. Hudson J. I. Jensen J. E. McCarthy J. Roberts J. L. Prescot A. P.. (2010). Rapid enhancement of glutamatergic neurotransmission in bipolar depression following treatment with riluzole. Neuropsychopharmacology 35, 834–846. 10.1038/npp.2009.191 19956089 PMC3055603

[B27] Buchsbaum M. S. Wu J. Siegel B. V. Hackett E. Trenary M. Abel L.. (1997). Effect of sertraline on regional metabolic rate in patients with affective disorder. Biol. Psychiatry 41, 15–22. 10.1016/s0006-3223(96)00097-2 8988791

[B29] Cahoy J. D. Emery B. Kaushal A. Foo L. C. Zamanian J. L. Christopherson K. S.. (2008). A transcriptome database for astrocytes, neurons and oligodendrocytes: a new resource for understanding brain development and function. J. Neurosci. 28, 264–278. 10.1523/JNEUROSCI.4178-07.2008 18171944 PMC6671143

[B30] Carrey N. J. MacMaster F. P. Gaudet L. Schmidt M. H. (2007). Striatal creatine and glutamate/glutamine in attention-deficit/hyperactivity disorder. J. Child Adolesc. Psychopharmacol. 17, 11–17. 10.1089/cap.2006.0008 17343550

[B31] Catches J. S. Xu J. Contractor A. (2012). Genetic ablation of the GluK4 kainate receptor subunit causes anxiolytic and antidepressant-like behavior in mice. Behav. Brain Res. 228, 406–414. 10.1016/j.bbr.2011.12.026 22203159 PMC3268896

[B32] Chen Y. Peng L. Zhang X. Stolzenburg J. U. Hertz L. (1995). Further evidence that fluoxetine interacts with a 5-HT_2C_ receptor in glial cells. Brain Res. Bull. 38, 153–159. 10.1016/0361-9230(95)00082-p 7583341

[B33] Choi D. S. Maroteaux L. (1996). Immunohistochemical localisation of the serotonin 5-HT_2B_ receptor in mouse gut, cardiovascular system and brain. FEBS. Lett. 391, 45–51. 10.1016/0014-5793(96)00695-3 8706927

[B34] Chowdhury G. M. Banasr M. de Graaf R. A. Rothman D. L. Behar K. L. Sanacora G. (2008). Chronic riluzole treatment increases glucose metabolism in rat prefrontal cortex and hippocampus. J. Cereb. Blood Flow Metab. 28, 1892–1897. 10.1038/jcbfm.2008.78 18628780 PMC2739056

[B35] Chowdhury G. M. Behar K. L. Cho W. Thomas M. A. Rothman D. L. Sanacora G. (2012). ^1^H-[^13^C]-nuclear magnetic resonance spectroscopy measures of ketamine’s effect on amino acid neurotransmitter metabolism. Biol. Psychiatry 71, 1022–1025. 10.1016/j.biopsych.2011.11.006 22169441 PMC3660962

[B36] Chowdhury G. M. Jiang L. Rothman D. L. Behar K. L. (2014). The contribution of ketone bodies to basal and activity-dependent neuronal oxidation in vivo. J. Cereb. Blood Flow Metab. 34, 1233–1242. 10.1038/jcbfm.2014.77 24780902 PMC4083391

[B37] Cohen M. L. Fludzinski L. A. (1987). Contractile serotonergic receptor in rat stomach fundus. J. Pharmacol. Exp. Ther. 243, 264–269. 3668856

[B38] Cruz N. F. Ball K. K. Dienel G. A. (2007). Functional imaging of focal brain activation in conscious rats: impact of [(14)C]glucose metabolite spreading and release. J. Neurosci. Res. 85, 3254–3266. 10.1002/jnr.22077 17265468

[B39] Cryan J. F. Lucki I. J. (2000). Antidepressant-like behavioral effects mediated by 5-Hydroxytryptamine(2C) receptors. Pharmacol. Exp. Ther. 295, 1120–1126. 11082448

[B40] Daub H. Wallasch C. Lankenau A. Herrlich A. Ullrich A. (1997). Signal characteristics of G protein-transactivated EGF receptor. EMBO J. 16, 7032–7044. 10.1093/emboj/16.23.7032 9384582 PMC1170306

[B42] Deecher D. C. Wilcox B. D. Dave V. Rossman P. A. Kimelberg H. K. (1993). Detection of 5-hydroxytryptamine2 receptors by radioligand binding, northern blot analysis and Ca^2+^ responses in rat primary astrocyte cultures. J. Neurosci. Res. 35, 246–256. 10.1002/jnr.490350304 8394435

[B43] Delorme R. Krebs M. O. Chabane N. Roy I. Millet B. Mouren-Simeoni M. C.. (2004). Frequency and transmission of glutamate receptors GRIK2 and GRIK3 polymorphisms in patients with obsessive compulsive disorder. Neuroreport 15, 699–702. 10.1097/00001756-200403220-00025 15094479

[B44] Denton R. M. McCormack J. G. (1990). Ca^2+^ as a second messenger within mitochondria of the heart and other tissues. Annu. Rev. Physiol. 52, 451–466. 10.1146/annurev.physiol.52.1.451 2184763

[B41] De Sarno P. Li X. Jope R. S. (2002). Regulation of Akt and glycogen synthase kinase-3 beta phosphorylation by sodium valproate and lithium. Neuropharmacology 43, 1158–1164. 10.1016/s0028-3908(02)00215-0 12504922

[B45] Descarries L. Riad M. (2012). Effects of the antidepressant fluoxetine on the subcellular localization of 5-HT_1A_ receptors and SERT. Philos. Trans. R. Soc. Lond. B Biol. Sci. 367, 2416–2425. 10.1098/rstb.2011.0361 22826342 PMC3405674

[B46] Diaz S. L. Doly S. Narboux-Nême N. Fernández S. Mazot P. Banas S. M.. (2012). 5-HT(2B) receptors are required for serotonin-selective antidepressant actions. Mol. Psychiatry 17, 154–163. 10.1038/mp.2011.159 22158014 PMC3381222

[B47] Du T. Li B. Liu S. Zang P. Prevot V. Hertz L.. (2009). ERK phosphorylation in intact, adult brain by alpha(2)-adrenergic transactivation of EGF receptors. Neurochem. Int. 55, 593–600. 10.1016/j.neuint.2009.05.016 19501623

[B48] Du T. Liang C. Li B. Hertz L. Peng L. (2014). Chronic fluoxetine administration increases expression of the L-channel gene Cav_1.2_ in astrocytes from the brain of treated mice and in culture and augments K(+)-induced increase in [Ca(2+)]_i_. Cell Calcium 55, 166–174. 10.1016/j.ceca.2014.01.002 24513410

[B49] Duarte J. M. Gruetter R. (2013). Glutamatergic and GABAergic energy metabolism measured in the rat brain by (13) C NMR spectroscopy at 14.1 T. J. Neurochem. 126, 579–590. 10.1111/jnc.12333 23745684

[B50] Duran J. Saez I. Gruart A. Guinovart J. J. Delgado-García J. M. (2013). Impairment in long-term memory formation and learning-dependent synaptic plasticity in mice lacking glycogen synthase in the brain. J. Cereb. Blood Flow Metab. 33, 550–556. 10.1038/jcbfm.2012.200 23281428 PMC3618391

[B51] Eid T. Behar K. Dhaher R. Bumanglag A. V. Lee T.-S. (2012). Roles of glutamine synthetase inhibition in epilepsy. Neurochem. Res. 37, 2339–2350. 10.1007/s11064-012-0766-5 22488332 PMC3731630

[B52] Ellfolk M. Malm H. (2010). Risks associated with in utero and lactation exposure to selective serotonin reuptake inhibitors (SSRIs). Reprod. Toxicol. 30, 249–260. 10.1016/j.reprotox.2010.04.015 20447455

[B53] Embi N. Rylatt D. B. Cohen P. (1980). Glycogen synthase kinase-3 from rabbit skeletal muscle. Separation from cyclic-AMP-dependent protein kinase and phosphorylase kinase. Eur. J. Biochem. 107, 519–527. 10.1111/j.1432-1033.1980.tb06059.x 6249596

[B54] Englander M. T. Dulawa S. C. Bhansali P. Schmauss C. (2005). How stress and fluoxetine modulate serotonin 2C receptor pre-mRNA editing. J. Neurosci. 25, 648–651. 10.1523/jneurosci.3895-04.2005 15659601 PMC6725319

[B55] Eom T. Y. Jope R. S. (2009). Blocked inhibitory serine-phosphorylation of glycogen synthase kinase-3alpha/beta impairs in vivo neural precursor cell proliferation. Biol. Psychiatry 66, 494–502. 10.1016/j.biopsych.2009.04.015 19520363 PMC2746934

[B56] Fang C. H. Li B. G. James J. H. King J. K. Evenson A. R. Warden G. D.. (2005). Protein breakdown in muscle from burned rats is blocked by insulin-like growth factor i and glycogen synthase kinase-3beta inhibitors. Endocrinology 146, 3141–3149. 10.1210/en.2004-0869 15802492

[B57] Fava M. Rosenbaum J. F. (1991). Suicidality and fluoxetine: is there a relationship? J. Clin. Psychiatry 52, 108–111. 2005073

[B58] Felder C. C. Kanterman R. Y. Ma A. L. Axelrod J. (1990). Serotonin stimulates phospholipase A2 and the release of arachidonic acid in hippocampal neurons by a type 2 serotonin receptor that is independent of inositolphospholipid hydrolysis. Proc. Natl. Acad. Sci. U S A 87, 2187–2191. 10.1073/pnas.87.6.2187 2315313 PMC53651

[B59] Freitas A. E. Machado D. G. Budni J. Neis V. B. Balen G. O. Lopes M. W.. (2013). Fluoxetine modulates hippocampal cell signaling pathways implicated in neuroplasticity in olfactory bulbectomized mice. Behav. Brain Res. 237, 176–184. 10.1016/j.bbr.2012.09.035 23018126

[B60] Fuller R. W. Wong D. T. (1977). Inhibition of serotonin reuptake. Fed. Proc. 36, 2154–2158. 326579

[B61] Gaspers L. D. Thomas A. P. (2008). Calcium-dependent activation of mitochondrial metabolism in mammalian cells. Methods 46, 224–232. 10.1016/j.ymeth.2008.09.012 18854213 PMC2640951

[B62] Ge W. P. Miyawaki A. Gage F. H. Jan Y. N. Jan L. Y. (2012). Local generation of glia is a major astrocyte source in postnatal cortex. Nature 484, 376–380. 10.1038/nature10959 22456708 PMC3777276

[B63] Gibbs M. E. Hertz L. (2014). Serotonin mediation of early memory formation via 5-HT_2B_ receptor-induced glycogenolysis in the day-old chick. Front. Pharmacol. 5:54. 10.3389/fphar.2014.00054 24744730 PMC3978258

[B64] Gibbs M. E. Hutchinson D. S. (2012). Rapid turnover of glycogen in memory formation. Neurochem. Res. 37, 2456–2463. 10.1007/s11064-012-0805-2 22664636

[B65] Gibbs M. E. Hutchinson D. Hertz L. (2008). Astrocytic involvement in learning and memory consolidation. Neurosci. Biobehav. Rev. 32, 927–944. 10.1016/j.neubiorev.2008.02.001 18462796

[B66] Gibbs M. E. Lloyd H. G. Santa T. Hertz L. (2007). Glycogen is a preferred glutamate precursor during learning in 1-day-old chick: biochemical and behavioral evidence. J. Neurosci. Res. 85, 3326–3333. 10.1002/jnr.21307 17455305

[B67] Gosselin R. D. Gibney S. O’Malley D. Dinan T. G. Cryan J. F. (2009). Region specific decrease in glial fibrillary acidic protein immunoreactivity in the brain of a rat model of depression. Neuroscience 159, 915–925. 10.1016/j.neuroscience.2008.10.018 19000745

[B68] Gould T. D. Picchini A. M. Einat H. Manji H. K. (2006). Targeting glycogen synthase kinase-3 in the CNS: implications for the development of new treatments for mood disorders. Curr. Drug Targets 7, 1399–1409. 10.2174/1389450110607011399 17100580

[B69] Griffin L. D. Mellon S. H. (1999). Selective serotonin reuptake inhibitors directly alter activity of neurosteroidogenic enzymes. Proc. Natl. Acad. Sci. U S A 96, 13512–13517. 10.1073/pnas.96.23.13512 10557352 PMC23979

[B70] Guirado R. Sanchez-Matarredona D. Varea E. Crespo C. Blasco-Ibáñez J. M. Nacher J. (2012). Chronic fluoxetine treatment in middle-aged rats induces changes in the expression of plasticity-related molecules and in neurogenesis. BMC Neurosci. 13:5. 10.1186/1471-2202-13-5 22221403 PMC3278353

[B71] Gurevich I. Tamir H. Arango V. Dwork A. J. Mann J. J. Schmauss C. (2002). Altered editing of serotonin 2C receptor pre-mRNA in the prefrontal cortex of depressed suicide victims. Neuron 34, 349–356. 10.1016/s0896-6273(02)00660-8 11988167

[B72] Halestrap A. P. (1989). The regulation of the matrix volume of mammalian mitochondria in vivo and in vitro and its role in the control of mitochondrial metabolism. Biochim. Biophys. Acta. 973, 355–382. 10.1016/s0005-2728(89)80378-0 2647140

[B73] Hashimoto K. Sawa A. Iyo M. (2007). Increased levels of glutamate in brains of patients with mood disorders. Biol. Psychiatry 25, 1310–1316. 10.1016/j.biopsych.2007.03.017 17574216

[B74] Hertz L. (2011). Astrocytic energy metabolism and glutamate formation–relevance for ^13^C-NMR spectroscopy and importance of cytosolic/mitochondrial trafficking. Magn. Reson. Imaging 29, 1319–1329. 10.1016/j.mri.2011.04.013 21820830

[B75] Hertz L. (2013). The Glutamate-Glutamine (GABA) cycle: importance of late postnatal development and potential reciprocal interactions between biosynthesis and degradation. Front. Endocrinol. (Lausanne) 4:59. 10.3389/fendo.2013.00059 23750153 PMC3664331

[B77] Hertz L. Baldwin F. Schousboe A. (1979). Serotonin receptors on astrocytes in primary cultures: effects of methysergide and fluoxetine. Can. J. Physiol. Pharmacol. 57, 223–226. 10.1139/y79-034 317577

[B78] Hertz L. Gerkau N. J. Xu J. Durry S. Song D. Rose C.. (2014b). Roles of astrocytic Na^+^,K^+^-ATPase and glycogenolysis for K^+^ homeostasis in mammalian brain. J. Neurosci. Res. [Epub ahead of print]. 10.1002/jnr.23499 25352321

[B79] Hertz L. Li B. Song D. Ren J. Dong L. Chen Y. (2012). Astrocytes as a 5-HT_2B_-mediated, SERT-independent SSRI target, slowly altering depression-associated genes and functions. Curr. Signal Transduct. Ther. 7, 65–80 10.2174/1574362799278154

[B80] Hertz L. Lovatt D. Goldman S. A. Nedergaard M. (2010). Adrenoceptors in brain: cellular gene expression and effects on astrocytic metabolism and [Ca(2+)]_i_. Neurochem. Int. 57, 411–420. 10.1016/j.neuint.2010.03.019 20380860 PMC2934885

[B81] Hertz L. Song D. Li B. Du T. Xu J. Gu L. (2014a). Signal transduction in astrocytes during chronic or acute treatment with drugs (SSRIs; anti-bipolar drugs; GABA-ergic drugs; benzodiazepines) ameliorating mood disorders. J. Signal Transduct. 2014:593934 10.1155/2014/593934 24707399 PMC3953578

[B82] Hertz L. Xu J. Song D. Du T. Li B. Yan E.. (2015). Astrocytic glycogenolysis: mechanisms and functions. Metab. Brain Dis. 30, 317–333. 10.1007/s11011-014-9536-1 24744118

[B83] Hertz L. Xu J. Song D. Du T. Yan E. Peng L. (2013a). Brain glycogenolysis, adrenoceptors, pyruvate carboxylase, Na(+),K(+)-ATPase and Marie E. Gibbs’ pioneering learning studies. Front. Integr. Neurosci. 7:20. 10.3389/fnint.2013.00020 23565080 PMC3615183

[B84] Hertz L. Xu J. Song D. Yan E. Gu L. Peng L. (2013b). Astrocytic and neuronal accumulation of elevated extracellular K(+) with a 2/3 K(+)/Na(+) flux ratio-consequences for energy metabolism, osmolarity and higher brain function. Front. Comput. Neurosci. 7:114. 10.3389/fncom.2013.00114 23986689 PMC3749512

[B85] Hertz L. Zielke H. R. (2004). Astrocytic control of glutamatergic activity: astrocytes as stars of the show. Trends Neurosci. 27, 735–743. 10.1016/j.tins.2004.10.008 15541514

[B86] Huang R. Chen Y. Yu A. C. Hertz L. (2000). Dexmedetomidine-induced stimulation of glutamine oxidation in astrocytes: a possible mechanism for its neuroprotective activity. J. Cereb. Blood Flow Metab. 20, 895–898. 10.1097/00004647-200006000-00001 10894172

[B87] Huang R. Hertz L. (1995). Noradrenaline-induced stimulation of glutamine metabolism in primary cultures of astrocytes. J. Neurosci. Res. 41, 677–683. 10.1002/jnr.490410514 7563248

[B88] Hyder F. Rothman D. L. (2012). Quantitative fMRI and oxidative neuroenergetics. Neuroimage 62, 985–994. 10.1016/j.neuroimage.2012.04.027 22542993 PMC3389300

[B89] Jenck F. Bös M. Wichmann J. Stadler H. Martin J. R. Moreau J. L. (1998). The role of 5-HT_2C_ receptors in affective disorders. Expert Opin. Investig. Drugs 7, 1587–1599. 10.1517/13543784.7.10.1587 15991903

[B90] Johnson D. A. Ingram C. D. Grant E. J. Craighead M. Gartside S. E. (2009). Glucocorticoid receptor antagonism augments fluoxetine-induced downregulation of the 5-HT transporter. Neuropsychopharmacology 34, 399–409. 10.1038/npp.2008.70 18496518

[B91] Kanner A. M. (2014). Is depression associated with an increased risk of treatment-resistant epilepsy? Research strategies to investigate this question. Epilepsy Behav. 38, 3–7. 10.1016/j.yebeh.2014.06.027 25260238

[B92] Kennedy S. H. Evans K. R. Krüger S. Mayberg H. S. Meyer J. H. McCann S.. (2001). Changes in regional brain glucose metabolism measured with positron emission tomography after paroxetine treatment of major depression. Am. J. Psychiatry 158, 899–905. 10.1176/appi.ajp.158.987.899 11384897

[B93] Kennett G. A. Wood M. D. Bright F. Trail B. Riley G. Holland V.. (1997). SB 242084, a selective and brain penetrant 5-HT_2C_ receptor antagonist. Neuropharmacology 36, 609–620. 10.1016/s0028-3908(97)00038-5 9225286

[B94] Kimbrell T. A. Ketter T. A. George M. S. Little J. T. Benson B. E. Willis M. W.. (2002). Regional cerebral glucose utilization in patients with a range of severities of unipolar depression. Biol. Psychiatry 51, 237–252. 10.1016/S0006-3223(01)01216-1 11839367

[B95] Kiyokage E. Toida K. Suzuki-Yamamoto T. Ishimura K. (2014). Cellular localization of 5α-reductase in the rat cerebellum. J. Chem. Neuroanat. 59–60, 8–16 10.1016/j.jchemneu.2014.04.002 24810015

[B96] Kong E. K. Peng L. Chen Y. Yu A. C. H. Hertz L. (2002). Up-regulation of 5-HT_2B_ receptor density and receptor-mediated glycogenolysis in mouse astrocytes by long-term fluoxetine administration. Neurochem. Res. 27, 113–120. 10.1023/A:1014862808126 11930908

[B203] Kraus J. E. Horrigan J. P. Carpenter D. J. Fong R. Barrett P. S. Davies J. T. (2010). Clinical features of patients with treatment-emergent suicidal behavior following initiation of paroxetine therapy. J. Affect. Disord. 120, 40–47. 10.1016/j.jad.2009.04.004 19439363

[B97] Kursar J. D. Nelson D. L. Wainscott D. B. Baez M. (1994). Molecular cloning, functional expression and mRNA tissue distribution of the human 5-hydroxytryptamine2B receptor. Mol. Pharmacol. 46, 227–234. 8078486

[B98] Langer S. Z. Galzin A. M. Lee C. R. Schoemaker H. (1986). Antidepressant-binding sites in brain and platelets. Ciba Found. Symp. 123, 3–29. 3816412 10.1002/9780470513361.ch2

[B99] Lanz B. Gruetter R. Duarte J. M. (2013). Metabolic flux and compartmentation analysis in the brain in vivo. Front. Endocrinol. (Lausanne) 4:156. 10.3389/fendo.2013.00156 24194729 PMC3809570

[B100] Launay J. M. Schneider B. Loric S. Da Prada M. Kellermann O. (2006). Serotonin transport and serotonin transporter-mediated antidepressant recognition are controlled by 5-HT_2B_ receptor signaling in serotonergic neuronal cells. FASEB J. 20, 1843–1854. 10.1096/fj.06-5724com 16940156

[B101] Lautens L. L. Chiou X. G. Sharp J. D. Young W. S. 3rd Sprague D. L. Ross L. S.. (1998). Cytosolic phospholipase A2 (cPLA_2_) distribution in murine brain and functional studies indicate that cPLA_2_ does not participate in muscarinic receptor-mediated signaling in neurons. Brain Res. 809, 18–30. 10.1016/s0006-8993(98)00806-3 9795110

[B103] Lebon V. Petersen K. F. Cline G. W. Shen J. Mason G. F. Dufour S.. (2002). Astroglial contribution to brain energy metabolism in humans revealed by ^13^C nuclear magnetic resonance spectroscopy: elucidation of the dominant pathway for neurotransmitter glutamate repletion and measurement of astrocytic oxidative metabolism. J. Neurosci. 22, 1523–1531. 11880482 10.1523/JNEUROSCI.22-05-01523.2002 PMC2995528

[B104] Lee Y. Son H. Kim G. Kim S. Lee D. H. Roh G. S.. (2013). Glutamine deficiency in the prefrontal cortex increases depressive-like behaviours in male mice. J. Psychiatry Neurosci. 38, 183–191. 10.1503/jpn.120024 23031251 PMC3633711

[B102] Le Poul E. Boni C. Hanoun N. Laporte A. M. Laaris N. Chauveau J.. (2000). Differential adaptation of brain 5-HT_1A_ and 5-HT_1B_ receptors and 5-HT transporter in rats treated chronically with fluoxetine. Neuropharmacology 39, 110–122. 10.1016/s0028-3908(99)00088-x 10665824

[B105] Lesch K. P. Aulakh C. S. Wolozin B. L. Tolliver T. J. Hill J. L. Murphy D. L. (1993). Regional brain expression of serotonin transporter mRNA and its regulation by reuptake inhibiting antidepressants. Brain Res. Mol. Brain Res. 17, 31–35. 10.1016/0169-328x(93)90069-2 8381906

[B106] Leysen D. C. (1999). Selective 5-HT_2C_ agonists as potential antidepressants. IDrugs 2, 109–120. 16160946

[B107] Li B. Dong L. Fu H. Wang B. Hertz L. Peng L. (2011b). Effects of chronic treatment with fluoxetine on receptor-stimulated increase of [Ca^2+^]_i_ in astrocytes mimic those of acute inhibition of TRPC1 channel activity. Cell Calcium 50, 42–53. 10.1016/j.ceca.2011.05.001 21640379

[B108] Li B. Dong L. Wang B. Cai L. Jiang N. Peng L. (2012). Cell type-specific gene expression and editing responses to chronic fluoxetine treatment in the in vivo mouse brain and their relevance for stress-induced anhedonia. Neurochem. Res. 37, 2480–2495. 10.1007/s11064-012-0814-1 22711334

[B109] Li B. Du T. Li H. Gu L. Zhang H. Huang J.. (2008b). Signalling pathways for transactivation by dexmedetomidine of epidermal growth factor receptors in astrocytes and its paracrine effect on neurons. Br. J. Pharmacol. 154, 191–203. 10.1038/bjp.2008.58 18311185 PMC2438992

[B110] Li B. Gu L. Hertz L. Peng L. (2013). Expression of nucleoside transporter in freshly isolated neurons and astrocytes from mouse brain. Neurochem. Res. 38, 2351–2358. 10.1007/s11064-013-1146-5 24026568

[B114] Li X. Jope R. S. (2010). Is glycogen synthase kinase-3 α central modulator in mood regulation? Neuropsychopharmacology 35, 2143–2154. 10.1038/npp.2010.105 20668436 PMC3055312

[B111] Li B. Zhang S. Li M. Hertz L. Peng L. (2009). Chronic treatment of astrocytes with therapeutically relevant fluoxetine concentrations enhances cPLA_2_ expression secondary to 5-HT_2B_-induced, transactivation-mediated ERK_1/2_ phosphorylation. Psychopharmacology (Berl) 207, 1–12. 10.1007/s00213-009-1631-3 19662385

[B112] Li B. Zhang S. Zhang H. Hertz L. Peng L. (2011a). Fluoxetine affects GluK2 editing, glutamate-evoked Ca(2+) influx and extracellular signal-regulated kinase phosphorylation in mouse astrocytes. J. Psychiatry Neurosci. 36, 322–338. 10.1503/jpn.100094 21320410 PMC3163648

[B113] Li B. Zhang S. Zhang H. Nu W. Cai L. Hertz L.. (2008a). Fluoxetine-mediated 5-HT_2B_ receptor stimulation in astrocytes causes EGF receptor transactivation and ERK phosphorylation. Psychopharmacology (Berl) 201, 443–458. 10.1007/s00213-008-1306-5 18758753

[B115] Li X. Zhu W. Roh M. S. Friedman A. B. Rosborough K. Jope R. S. (2004). In vivo regulation of glycogen synthase kinase-3beta (GSK3beta) by serotonergic activity in mouse brain. Neuropsychopharmacology 29, 1426–1431. 10.1038/sj.npp.1300439 15039769 PMC1986663

[B116] Lightowler S. Kennett G. A. Williamson I. J. Blackburn T. P. Tulloch I. F. (1994). Anxiolytic-like effect of paroxetine in a rat social interaction test. Pharmacol. Biochem. Behav. 49, 281–285. 10.1016/0091-3057(94)90422-7 7824539

[B117] Little J. T. Ketter T. A. Kimbrell T. A. Danielson A. Benson B. E. Willis M. W.. (1996). Venlafaxine or bupropion responders but not nonresponders show baseline prefrontal and paralimbic hypometabolism compared with controls. Psychopharmacol. Bull. 32, 629–635. 8993084

[B118] Little J. T. Ketter T. A. Kimbrell T. A. Dunn R. T. Benson B. E. Willis M. W.. (2005). Bupropion and venlafaxine responders differ in pretreatment regional cerebral metabolism in unipolar depression. Biol. Psychiatry 57, 220–228. 10.1016/j.biopsych.2004.10.033 15691522

[B119] Lovatt D. Sonnewald U. Waagepetersen H. S. Schousboe A. He W. Lin J. H.. (2007). The transcriptome and metabolic gene signature of protoplasmic astrocytes in the adult murine cortex. J. Neurosci. 27, 12255–12266. 10.1523/jneurosci.3404-07.2007 17989291 PMC6673251

[B120] Manev H. Uz T. Manev R. (2003). Glia as a putative target for antidepressant treatments. J. Affect. Disord. 75, 59–64. 10.1016/s0165-0327(02)00044-7 12781351

[B121] Mangia S. Giove F. Dinuzzo M. (2012). Metabolic pathways and activity-dependent modulation of glutamate concentration in the human brain. Neurochem. Res. 37, 2554–2561. 10.1007/s11064-012-0848-4 22846967 PMC3489977

[B122] Mangia S. Giove F. Dinuzzo M. (2013). K^+^ homeostasis in the brain: a new role for glycogenolysis. Neurochem. Res. 38, 470–471. 10.1007/s11064-012-0962-3 23292196

[B123] Martin J. R. Bös M. Jenck F. Moreau J. Mutel V. Sleight A. J.. (1998). 5-HT_2C_ receptor agonists: pharmacological characteristics and therapeutic potential. J. Pharmacol. Exp. Ther. 286, 913–924. 9694950

[B28] Mayberg H. S. Brannan S. K. Tekell J. L. Silva J. A. Mahurin R. K. McGinnis S.. (2000). Regional metabolic effects of fluoxetine in major depression: serial changes and relationship to clinical response. Biol. Psychiatry 48, 830–843. 10.1016/s0006-3223(00)01036-2 11063978

[B124] Mercier G. Lennon A. M. Renouf B. Dessouroux A. Ramaugé M. Courtin F.. (2004). MAP kinase activation by fluoxetine and its relation to gene expression in cultured rat astrocytes. J. Mol. Neurosci. 24, 207–216. 10.1385/jmn:24:2:207 15456934

[B125] Meyer J. H. Wilson A. A. Sagrati S. Hussey D. Carella A. Potter W. Z.. (2004). Serotonin transporter occupancy of five selective serotonin reuptake inhibitors at different doses: an [^11^C]DASB positron emission tomography study. Am. J. Psychiatry 161, 826–835. 10.1176/appi.ajp.161.5.826 15121647

[B126] Mitani H. Shirayama Y. Yamada T. Maeda K. Ashby C. R. Jr. Kawahara R. (2006). Correlation between plasma levels of glutamate, alanine and serine with severity of depression. Prog. Neuropsychopharmacol. Biol. Psychiatry 30, 1155–1158. 10.1016/j.pnpbp.2006.03.036 16707201

[B127] Muguruza C. Miranda-Azpiazu P. Díez-Alarcia R. Morentin B. González-Maeso J. Callado L. F.. (2014). Evaluation of 5-HT_2A_ and mGlu2/3 receptors in postmortem prefrontal cortex of subjects with major depressive disorder: effect of antidepressant treatment. Neuropharmacology 86, 311–318. 10.1016/j.neuropharm.2014.08.009 25150943 PMC12706166

[B128] Murakami M. Shimbara S. Kambe T. Kuwata H. Winstead M. V. Tischfield J. A.. (1998). The functions of five distinct mammalian phospholipase A2S in regulating arachidonic acid release. Type IIa and type V secretory phospholipase A2S are functionally redundant and act in concert with cytosolic phospholipase A2. J. Biol. Chem. 273, 14411–14423. 10.1074/jbc.273.23.14411 9603953

[B129] Niciu M. J. Henter I. D. Luckenbaugh D. A. Zarate C. A. Jr. Charney D. S. (2014). Glutamate receptor antagonists as fast-acting therapeutic alternatives for the treatment of depression: ketamine and other compounds. Annu. Rev. Pharmacol. Toxicol. 54, 119–139. 10.1146/annurev-pharmtox-011613-135950 24392693 PMC4089991

[B130] Nierenberg A. A. Farabaugh A. H. Alpert J. E. Gordon J. Worthington J. J. Rosenbaum J. F.. (2000). Timing of onset of antidepressant response with fluoxetine treatment. Am. J. Psychiatry 157, 1423–1428. 10.1176/appi.ajp.157.9.1423 10964858

[B131] Nissen-Meyer L. S. Chaudhry F. A. (2013). Protein Kinase C Phosphorylates the System N Glutamine Transporter SN1 (Slc38a3) and regulates its membrane trafficking and degradation. Front. Endocrinol. (Lausanne) 4:138. 10.3389/fendo.2013.00138 24106489 PMC3788335

[B132] Niswender C. M. Herrick-Davis K. Dilley G. E. Meltzer H. Y. Overholser J. C. Stockmeier C. A.. (2001). RNA editing of the human serotonin 5-HT_2C_ receptor. Alterations in suicide and implications for serotonergic pharmacotherapy. Neuropsychopharmacology 24, 478–491. 10.1016/s0893-133x(00)00223-2 11282248

[B135] Obel L. F. Müller M. S. Walls A. B. Sickmann H. M. Bak L. K. Waagepetersen H. S.. (2012). Brain glycogen-new perspectives on its metabolic function and regulation at the subcellular level. Front. Neuroenergetics 4:3. 10.3389/fnene.2012.00003 22403540 PMC3291878

[B133] O’Connor R. M. Pusceddu M. M. Dinan T. G. Cryan J. F. (2013). Impact of early-life stress, on group III mGlu receptor levels in the rat hippocampus: effects of ketamine, electroconvulsive shock therapy and fluoxetine treatment. Neuropharmacology 66, 236–241. 10.1016/j.neuropharm.2012.05.006 22609536

[B134] O’Leary O. F. Dinan T. G. Cryan J. F. (2014). Faster, better, stronger: towards new antidepressant therapeutic strategies. Eur. J. Pharmacol. [Epub ahead of print]. 10.1016/j.ejphar.2014.07.046 25092200

[B136] Oyagi A. Moriguchi S. Nitta A. Murata K. Oida Y. Tsuruma K.. (2011). Heparin-binding EGF-like growth factor is required for synaptic plasticity and memory formation. Brain Res. 1419, 97–104. 10.1016/j.brainres.2011.09.003 21945083

[B137] Pae C. U. Yu H. S. Kim J. J. Lee C. U. Lee S. J. Lee K. U.. (2004). BanI polymorphism of the cytosolic phospholipase A2 gene and mood disorders in the Korean population. Neuropsychobiology 49, 185–188. 10.1159/000077364 15118355

[B138] Palaiologos G. Hertz L. Schousboe A. (1989). Role of aspartate aminotransferase and mitochondrial dicarboxylate transport for release of endogenously and exogenously supplied neurotransmitter in glutamatergic neurons. Neurochem. Res. 14, 359–366. 10.1007/bf01000039 2569674

[B139] Patel A. B. de Graaf R. A. Mason G. F. Rothman D. L. Shulman R. G. Behar K. L. (2005). The contribution of GABA to glutamate/glutamine cycling and energy metabolism in the rat cortex in vivo. Proc. Natl. Acad. Sci. U S A 102, 5588–5593. 10.1073/pnas.0501703102 15809416 PMC556230

[B140] Patel A. B. de Graaf R. A. Rothman D. L. Behar K. L. (2015). Effects of GAT1 inhibtion by tiagabine on brain glutamate and GABA metabolism in the anesthetized rat in vivo. J. Neurosci. Res. 10.1002/jnr.23548 PMC4441585 25663257

[B141] Peavy R. D. Chang M. S. Sanders-Bush E. Conn P. J. (2001). Metabotropic glutamate receptor 5-induced phosphorylation of extracellular signal-regulated kinase in astrocytes depends on transactivation of the epidermal growth factor receptor. J. Neurosci. 21, 9619–9628. 11739572 10.1523/JNEUROSCI.21-24-09619.2001 PMC6763045

[B142] Peca S. Carnì M. Di Bonaventura C. Aprile T. Hagberg G. E. Giallonardo A. T.. (2010). Metabolic correlatives of brain activity in a FOS epilepsy patient. NMR Biomed. 23, 170–178. 10.1002/nbm.1439 19839013

[B143] Peng L. (2004). “Transactivation in astrocytes as a novel mechanism of neuroprotection,” in Non-neuronal Cells of the Nervous System: Function and Dysfunction, ed Hertz L. (Amsterdam: Elsevier), 503–518.

[B144] Peng L. Gu L. Li B. Hertz L. (2014). Fluoxetine and all other SSRIs are 5-HT_2B_ Agonists–Importance for their therapeutic effects. Curr. Neuropharmacol. 12, 365–379. 10.2174/1570159x12666140828221720 25342944 PMC4207076

[B145] Pinna G. Costa E. Guidotti A. (2009). SSRIs act as selective brain steroidogenic stimulants (SBSSs) at low doses that are inactive on 5-HT reuptake. Curr. Opin. Pharmacol. 9, 24–30. 10.1016/j.coph.2008.12.006 19157982 PMC2670606

[B146] Plenge P. (1976). Acute lithium effects on rat brain glucose metabolism-in vivo. Int. Pharmacopsychiatry 11, 84–92. 939673 10.1159/000468216

[B147] Plenge P. (1982). Lithium effects on rat brain glucose metabolism in vivo. Effects after administration of lithium by various routes. Psychopharmacology (Berl) 77, 348–355. 10.1007/bf00432769 6813896

[B148] Polter A. Beurel E. Yang S. Garner R. Song L. Miller C. A.. (2010). Deficiency in the inhibitory serine-phosphorylation of glycogen synthase kinase-3 increases sensitivity to mood disturbances. Neuropsychopharmacology 35, 1761–1774. 10.1038/npp.2010.43 20357757 PMC2891528

[B149] Polter A. M. Yang S. Jope R. S. Li X. (2012). Functional significance of glycogen synthase kinase-3 regulation by serotonin. Cell Signal. 24, 265–271. 10.1016/j.cellsig.2011.09.009 21946431 PMC3205250

[B150] Popik P. (1999). Preclinical pharmacology of citalopram. J. Clin. Psychopharmacol. 19, 4S–22S. 10.1097/00004714-199910001-00002 10507505

[B151] Porter R. H. Benwell K. R. Lamb H. Malcolm C. S. Allen N. H. Revell D. F.. (1999). Functional characterization of agonists at recombinant human 5-HT_2A_, 5-HT_2B_ and 5-HT_2C_ receptors in CHO-K1 cells. Br. J. Pharmacol. 128, 13–20. 10.1038/sj.bjp.0702751 10498829 PMC1571597

[B152] Pugh K. R. Frost S. J. Rothman D. L. Hoeft F. Del Tufo S. N. Mason G. F.. (2014). Glutamate and choline levels predict individual differences in reading ability in emergent readers. J. Neurosci. 34, 4082–4089. 10.1523/JNEUROSCI.3907-13.2014 24623786 PMC3951703

[B153] Qu Y. Chang L. Klaff J. Seemann R. Rapoport S. I. (2003). Imaging brain phospholipase A2-mediated signal transduction in response to acute fluoxetine administration in unanesthetized rats. Neuropsychopharmacology 28, 1219–1226. 10.1038/sj.npp.1300177 12784122

[B154] Rajkowska G. Stockmeier C. A. (2013). Astrocyte pathology in major depressive disorder: insights from human postmortem brain tissue. Curr. Drug Targets 14, 1225–1236. 10.2174/13894501113149990156 23469922 PMC3799810

[B155] Rao J. S. Ertley R. N. Lee H. J. Rapoport S. I. Bazinet R. P. (2006). Chronic fluoxetine upregulates activity, protein and mRNA levels of cytosolic phospholipase A2 in rat frontal cortex. Pharmacogenomics J. 6, 413–420. 10.1038/sj.tpj.6500391 16636684

[B156] Rapoport S. I. (2008). Brain arachidonic and docosahexaenoic acid cascades are selectively altered by drugs, diet and disease. Prostaglandins Leukot. Essent. Fatty Acids 79, 153–156. 10.1016/j.plefa.2008.09.010 18973997 PMC4576349

[B157] Rasgon N. L. Kenna H. A. Geist C. Small G. Silverman D. (2008). Cerebral metabolic patterns in untreated postmenopausal women with major depressive disorder. Psychiatry Res. 164, 77–80. 10.1016/j.pscychresns.2007.12.006 18707852

[B158] Rieckmann N. Kronish I. M. Shapiro P. A. Whang W. Davidson K. W. (2013). Serotonin reuptake inhibitor use, depression and long-term outcomes after an acute coronary syndrome: a prospective cohort study. JAMA Intern. Med. 173, 1150–1151. 10.1001/jamainternmed.2013.910 23699784 PMC3718306

[B159] Rosenzweig-Lipson S. Sabb A. Stack G. Mitchell P. Lucki I. Malberg J. E.. (2007). Antidepressant-like effects of the novel, selective, 5-HT_2C_ receptor agonist WAY-163909 in rodents. Psychopharmacology (Berl) 192, 159–170. 10.1007/s00213-007-0710-6 17297636

[B160] Roth B. L. Willins D. L. Kristiansen K. Kroeze W. K. (1998). 5-Hydroxytryptamine2-family receptors (5-hydroxytryptamine2A, 5-hydroxytryptamine2B, 5-hydroxytryptamine2C): where structure meets function. Pharmacol. Ther. 79, 231–257. 10.1016/s0163-7258(98)00019-9 9776378

[B161] Rothstein J. D. Dykes-Hoberg M. Pardo C. A. Bristol L. A. Jin L. Kunci R. W.. (1996). Knockout of glutamate transporters reveals a major role for astroglial transport in excitotoxicity and clearance of glutamate. Neuron 16, 675–686. 10.1016/s0896-6273(00)80086-0 8785064

[B162] Rubio F. J. Ampuero E. Sandoval R. Toledo J. Pancetti F. Wyneken U. (2013). Long-term fluoxetine treatment induces input-specific LTP and LTD impairment and structural plasticity in the CA1 hippocampal subfield. Front. Cell. Neurosci. 7:66. 10.3389/fncel.2013.00066 23675317 PMC3648695

[B163] Rutter G. A. Burnett P. Rizzuto R. Brini M. Murgia M. Pozzan T.. (1996). Subcellular imaging of intramitochondrial Ca^2+^ with recombinant targeted aequorin: significance for the regulation of pyruvate dehydrogenase activity. Proc. Natl. Acad. Sci. U S A 93, 5489–5494. 10.1073/pnas.93.11.5489 8643602 PMC39273

[B164] Sampaio A. S. Fagerness J. Crane J. Leboyer M. Delorme R. Pauls D. L.. (2011). Association between polymorphisms in GRIK2 gene and obsessive-compulsive disorder: a family-based study. CNS Neurosci. Ther. 17, 141–147. 10.1111/j.1755-5949.2009.00130.x 20370803 PMC6493828

[B165] Sanacora G. Banasr M. (2013). From pathophysiology to novel antidepressant drugs: glial contributions to the pathology and treatment of mood disorders. Biol. Psychiatry 73, 1172–1179. 10.1016/j.biopsych.2013.03.032 23726152 PMC3688253

[B166] Sanacora G. Gueorguieva R. Epperson C. N. Wu Y. T. Appel M. Rothman D. L.. (2004). Subtype-specific alterations of gamma-aminobutyric acid and glutamate in patients with major depression. Arch. Gen. Psychiatry 61, 705–713. 10.1001/archpsyc.61.7.705 15237082

[B167] Sanacora G. Kendell S. F. Levin Y. Simen A. A. Fenton L. R. Coric V.. (2007). Preliminary evidence of riluzole efficacy in antidepressant-treated patients with residual depressive symptoms. Biol. Psychiatry 61, 822–825. 10.1016/j.biopsych.2006.08.037 17141740 PMC2754299

[B168] Sanacora G. Mason G. F. Rothman D. L. Krystal J. H. (2002). Increased occipital cortex GABA concentrations in depressed patients after therapy with selective Serotonin Reuptake inhibitors. Am. J. Psychiatry 159, 663–665. 10.1176/appi.ajp.159.4.663 11925309

[B169] Sanacora G. Treccani G. Popoli M. (2012). Towards a glutamate hypothesis of depression an emerging frontier of neuropsychopharmacology for mood disorders. Neuropharmacology 62, 63–77. 10.1016/j.neuropharm.2011.07.036 21827775 PMC3205453

[B170] Sands S. A. Reisman S. A. Enna S. J. (2004). Effect of antidepressants on GABA(B) receptor function and subunit expression in rat hippocampus. Biochem. Pharmacol. 68, 1489–1495. 10.1016/j.bcp.2004.07.027 15451391

[B171] Sarkar A. Chachra P. Vaidya V. A. (2014). Postnatal fluoxetine-evoked anxiety is prevented by Concomitant 5-HT(2A/C) receptor blockade and mimicked by postnatal 5-HT(2A/C) receptor stimulation. Biol. Psychiatry 76, 858–868. 10.1016/j.biopsych.2013.11.005 24315410

[B172] Schaeffer E. L. Gattaz W. F. (2008). Cholinergic and glutamatergic alterations beginning at the early stages of Alzheimer disease: participation of the phospholipase A2 enzyme. Psychopharmacology (Berl) 198, 1–27. 10.1007/s00213-008-1092-0 18392810

[B173] Schipke C. G. Heuser I. Peters O. (2011). Antidepressants act on glial cells: SSRIs and serotonin elicit astrocyte calcium signaling in the mouse prefrontal cortex. J. Psychiatr. Res. 45, 242–248. 10.1016/j.jpsychires.2010.06.005 20619420

[B174] Schmauss C. Zimnisky R. Mehta M. Shapiro L. P. (2010). The roles of phospholipase C activation and alternative ADAR1 and ADAR2 pre-mRNA splicing in modulating serotonin 2C-receptor editing in vivo. RNA 16, 1779–1785. 10.1261/rna.2188110 20651031 PMC2924537

[B175] Schousboe A. (1971). Development of potassium effects on ion concentrations and indicator spaces in rat brain-cortex slices during postnatal ontogenesis. Exp. Brain Res. 15, 521–531. 10.1007/bf00236406 4673705

[B176] Schousboe A. Bak L. K. Waagepetersen H. S. (2013). Astrocytic control of Biosynthesis and turnover of the Neurotransmitters Glutamate and GABA. Front. Endocrinol. (Lausanne) 4:102. 10.3389/fendo.2013.00102 23966981 PMC3744088

[B177] Shaltiel G. Maeng S. Malkesman O. Pearson B. Schloesser R. J. Tragon T.. (2008). Evidence for the involvement of the kainate receptor subunit GluR6 (GRIK2) in mediating behavioral displays related to behavioral symptoms of mania. Mol. Psychiatry 13, 858–872. 10.1038/mp.2008.20 18332879 PMC2804880

[B178] Shank R. P. Bennett G. S. Freytag S. O. Campbell G. L. (1985). Pyruvate carboxylase: an astrocyte-specific enzyme implicated in the replenishment of amino acid neurotransmitter pools. Brain Res. 329, 364–367. 10.1016/0006-8993(85)90552-9 3884090

[B179] Sibson N. R. Dhankhar A. Mason G. F. Rothman D. L. Behar K. L. Shulman R. G. (1998). Stoichiometric coupling of brain glucose metabolism and glutamatergic neuronal activity. Proc. Natl. Acad. Sci. U S A 95, 316–321. 10.1073/pnas.95.1.316 9419373 PMC18211

[B180] Singh A. B. Bousman C. A. Ng C. H. Berk M. (2013). High impact child abuse may predict risk of elevated suicidality during antidepressant initiation. Aust. N Z J. Psychiatry 47, 1191–1195. 10.1177/0004867413510212 24280998

[B181] Sonnewald U. (2014). Glutamate synthesis has to be matched by its degradation- where do all the carbons go? J. Neurochem. 131, 399–406. 10.1111/jnc.12812 24989463

[B182] Sorg O. Pellerin L. Stolz M. Beggah S. Magistretti P. J. (1995). Adenosine triphosphate and arachidonic acid stimulate glycogenolysis in primary cultures of mouse cerebral cortical astrocytes. Neurosci. Lett. 188, 109–112. 10.1016/0304-3940(95)11410-x 7792053

[B183] Subbarao K. V. Hertz L. (1991). Stimulation of energy metabolism by alpha-adrenergic agonists in primary cultures of astrocytes. J. Neurosci. Res. 28, 399–405. 10.1002/jnr.490280312 1677429

[B184] Sublette M. E. Milak M. S. Hibbeln J. R. Freed P. J. Oquendo M. A. Malone K. M.. (2009). Plasma polyunsaturated fatty acids and regional cerebral glucose metabolism in major depression. Prostaglandins Leukot. Essent. Fatty Acids 80, 57–64. 10.1016/j.plefa.2008.11.004 19128951 PMC2712826

[B185] Sun G. Y. Xu J. Jensen M. D. Simonyi A. (2004). Phospholipase A2 in the central nervous system: implications for neurodegenerative diseases. J. Lipid. Res. 45, 205–213. 10.1194/jlr.r300016-jlr200 14657205

[B186] Trivedi M. H. Wisniewski S. R. Morris D. W. Fava M. Kurian B. T. Gollan J. K.. (2011). Concise associated symptoms tracking scale: a brief self-report and clinician rating of symptoms associated with suicidality. J. Clin. Psychiatry 72, 765–774. 10.4088/jcp.11m06840 21733477

[B187] Verkhratsky A. Nedergaard M. Hertz L. (2014). Why are astrocytes important? Neurochem. Res. [Epub ahead of print]. 10.1007/s11064-014-1403-2 25113122

[B188] Vialou V. Robison A. J. Laplant Q. C. Covington H. E. 3rd Dietz D. M. Ohnishi Y. N.. (2010). DeltaFosB in brain reward circuits mediates resilience to stress and antidepressant responses. Nat. Neurosci. 13, 745–752. 10.1038/nn.2551 20473292 PMC2895556

[B189] Vicente M. A. Zangrossi H. Jr. (2014). Involvement of 5-HT_2C_ and 5-HT_1A_ receptors of the basolateral nucleus of the amygdala in the anxiolytic effect of chronic antidepressant treatment. Neuropharmacology 79, 127–135. 10.1016/j.neuropharm.2013.11.007 24275045

[B190] Videbech P. (2000). PET measurements of brain glucose metabolism and blood flow in major depressive disorder: a critical review. Acta Psychiatr. Scand. 101, 11–20. 10.1034/j.1600-0447.2000.101001011.x 10674946

[B191] Webhofer C. Gormanns P. Reckow S. Lebar M. Maccarrone G. Ludwig T.. (2013). Proteomic and metabolomic profiling reveals time-dependent changes in hippocampal metabolism upon paroxetine treatment and biomarker candidates. J. Psychiatr. Res. 47, 289–298. 10.1016/j.jpsychires.2012.11.003 23207114

[B192] Wei S. Ong W. Y. Thwin M. M. Fong C. W. Farooqui A. A. Gopalakrishnakone P.. (2003). Group IIA secretory phospholipase A2 stimulates exocytosis and neurotransmitter release in pheochromocytoma-12 cells and cultured rat hippocampal neurons. Neuroscience 121, 891–898. 10.1016/s0306-4522(03)00525-6 14580939

[B193] Wong D. T. Bymaster F. P. (1995). Development of antidepressant drugs. Fluoxetine (Prozac) and other selective serotonin uptake inhibitors. Adv. Exp. Med. Biol. 363, 77–95. 10.1007/978-1-4615-1857-0_11 7618533

[B194] Xia M. Zhu Y. (2011). Signaling pathways of ATP-induced PGE2 release in spinal cord astrocytes are EGFR transactivation-dependent. Glia 59, 664–674. 10.1002/glia.21138 21294165

[B195] Xu J. Song D. Bai Q. Cai L. Hertz L. Peng L. (2014). Basic mechanism leading to stimulation of glycogenolysis by isoproterenol, EGF, elevated extracellular K^+^ concentrations, or GABA. Neurochem. Res. 39, 661–667 10.1007/s11064-014-1244-z 24500447

[B196] Xu J. Song D. Xue Z. Gu L. Hertz L. Peng L. (2013). Requirement of glycogenolysis for uptake of increased extracellular K^+^ in astrocytes: potential implications for K^+^ homeostasis and glycogen usage in brain. Neurochem. Res. 38, 472–485. 10.1007/s11064-012-0938-3 23232850

[B197] Yan E. Li B. Gu L. Hertz L. Peng L. (2013). Mechanisms for L-channel-mediated increase in [Ca(2+)](i) and its reduction by anti-bipolar drugs in cultured astrocytes combined with its mRNA expression in freshly isolated cells support the importance of astrocytic L-channels. Cell Calcium 54, 335–342. 10.1016/j.ceca.2013.08.002 24079970

[B198] Yu A. C. Drejer J. Hertz L. Schousboe A. (1983). Pyruvate carboxylase activity in primary cultures of astrocytes and neurons. J. Neurochem. 41, 1484–1487. 10.1111/j.1471-4159.1983.tb00849.x 6619879

[B199] Yu N. Martin J. L. Stella N. Magistretti P. J. (1993). Arachidonic acid stimulates glucose uptake in cerebral cortical astrocytes. Proc. Natl. Acad. Sci. U S A 90, 4042–4046. 10.1073/pnas.90.9.4042 8483920 PMC46442

[B200] Zhang S. Li B. Lovatt D. Xu J. Song D. Goldman S. A.. (2010). 5-HT_2B_ receptors are expressed on astrocytes from brain and in culture and are a chronic target for all five conventional ‘serotonin-specific reuptake inhibitors’. Neuron Glia Biol. 6, 113–125. 10.1017/s1740925x10000141 20846463

[B201] Zhang X. Peng L. Chen Y. Hertz L. (1993). Stimulation of glycogenolysis in astrocytes by fluoxetine, an antidepressant acting like 5-HT. Neuroreport 4, 1235–1238. 10.1097/00001756-199309000-00006 7693014

[B202] Zhou Z. Zhen J. Karpowich N. K. Law C. J. Reith M. E. Wang D. N. (2009). Antidepressant specificity of serotonin transporter suggested by three LeuT-SSRI structures. Nat. Struct. Mol. Biol. 16, 652–657. 10.1038/nsmb.1602 19430461 PMC2758934

